# The Lyme Disease Pathogen Has No Effect on the Survival of Its Rodent Reservoir Host

**DOI:** 10.1371/journal.pone.0118265

**Published:** 2015-02-17

**Authors:** Maarten J. Voordouw, Shelly Lachish, Marc C. Dolan

**Affiliations:** 1 Department of Biology, University of Pennsylvania, Philadelphia, Pennsylvania, United States of America; 2 Department of Zoology, Edward Grey Institute, University of Oxford, Oxford, United Kingdom; 3 Division of Vector-Borne Diseases, National Center for Enteric and Zoonotic Infectious Diseases, Centers for Disease Control and Prevention, Fort Collins, Colorado, United States of America; University of Kentucky College of Medicine, UNITED STATES

## Abstract

Zoonotic pathogens that cause devastating morbidity and mortality in humans may be relatively harmless in their natural reservoir hosts. The tick-borne bacterium *Borrelia burgdorferi* causes Lyme disease in humans but few studies have investigated whether this pathogen reduces the fitness of its reservoir hosts under natural conditions. We analyzed four years of capture-mark-recapture (CMR) data on a population of white-footed mice, *Peromyscus leucopus*, to test whether *B*. *burgdorferi* and its tick vector affect the survival of this important reservoir host. We used a multi-state CMR approach to model mouse survival and mouse infection rates as a function of a variety of ecologically relevant explanatory factors. We found no effect of *B*. *burgdorferi* infection or tick burden on the survival of *P*. *leucopus*. Our estimates of the probability of infection varied by an order of magnitude (0.051 to 0.535) and were consistent with our understanding of Lyme disease in the Northeastern United States. *B*. *burgdorferi* establishes a chronic avirulent infection in their rodent reservoir hosts because this pathogen depends on rodent mobility to achieve transmission to its sedentary tick vector. The estimates of *B*. *burgdorferi* infection risk will facilitate future theoretical studies on the epidemiology of Lyme disease.

## Introduction

There has been much recent interest in studying the ecology of wildlife and their zoonotic pathogens because these systems generate the vast majority of emerging infectious diseases that pose a threat to human health [1–3]. Initial studies of wildlife diseases tend to be qualitative in nature with an emphasis on characterizing the taxonomic identity of the relevant players: the pathogen, the intermediate and final hosts, and the vectors (in systems where the pathogen is transmitted by blood-feeding arthropods). The elucidation of the natural history of the zoonosis is generally followed by a growing interest in developing a more quantitative ecological description of the system. Such a quantitative description inevitably requires a thorough understanding of the parameters that govern the dynamics of the wildlife disease; parameters such as the infection rate, recovery rate, host survival rate, and pathogen-induced mortality rate (virulence). Estimates of these parameters are often difficult to obtain because they have to be sampled from populations of wild animals, which are by nature secretive, shy and refractory to observation. Fortunately, capture-mark-recapture (CMR) sampling methods and recent developments in the associated statistical approaches have given biologists a new set of tools for studying wildlife diseases.

CMR studies, where animals are individually marked and then followed through time, are the major methodology for studying the dynamics (birth rates, survival rates) of wildlife populations [4, 5]. With respect to the study of infectious diseases in wildlife, CMR statistical methods are particularly well suited for estimating whether the pathogen influences the survival rate as well as the capture or encounter rate of individuals from the host population. Many parasites can influence the behaviour of their hosts [6] with the result that infected and uninfected animals often have different capture or encounter rates [7–9]. CMR statistical methods allow scientists to model and thus control for possible differences in this detection bias between infected and uninfected hosts [10]. One particularly important development in the study of wildlife diseases has been the application of multi-state models, where individuals can transition between infected and uninfected states and which allow scientists to model the abiotic and biotic factors that influence the processes of infection and recovery. A number of recent studies have used this multistate model framework to study the dynamics of infectious diseases in wildlife populations [7–9, 11–15]. In this study, we used multistate models to study the effects of a tick-borne bacterium, *Borrelia burgdorferi* sensu stricto (s. s.), which causes human Lyme disease, on the survival of its natural rodent host, the white-footed mouse, *Peromyscus leucopus*.

Lyme disease is the most common vector-borne zoonosis in North America and is caused by the spirochete bacterium *Borrelia burgdorferi* s. s. and vectored by *Ixodes scapularis* ticks during blood feeding [16–18]. The disease is maintained in nature by alternating generations of immature ticks (nymphs and larvae) that feed on the same set of reservoir hosts. There is no vertical transmission of *B*. *burgdorferi* in the tick vector [19, 20]. Larval ticks acquire the pathogen by feeding on infected hosts and subsequently develop into infected nymphs. In the Northeastern United States, the two immature tick stages exhibit distinct seasonal activity patterns: the nymphs infect the reservoir hosts at the start of the summer and these infected hosts then transmit the pathogen to the larvae in late summer [21–23]. Reservoir hosts include ground-dwelling birds and small mammals [23, 24]. In the Northeastern US, the most important reservoir host in the Lyme disease life cycle is the white-footed mouse, *P*. *leucopus* [22, 25–29]. Serological surveys of wild *P*. *leucopus* populations have shown that over 90% of all mice are exposed to *B*. *burgdorferi* by the end of the transmission season [30]. Infected *P*. *leucopus* mice are believed to remain infected for life (i.e. no recovery) [22, 25, 31, 32] and are generally very efficient at transmitting the spirochetes to the ticks [31, 33–35]. Long-term fieldwork has shown that the population dynamics of *P*. *leucopus* influence the density of *B*. *burgdorferi*-infected nymphs [36]. Furthermore, host communities dominated by this host species have a high risk of Lyme disease [37]. *P*. *leucopus* thus plays an important role in the ecology of Lyme disease. To date, there is only one study that has investigated whether *B*. *burgdorferi* influences the fitness of *P*. *leucopus* mice in the field [32]. This study found that infected mice had a median lifespan (176 days) that was 12.8% longer than uninfected mice (156 days) although the difference was not statistically significant [32]. As the authors did not use CMR statistical methods to analyze their data, they were unable to separately estimate the effect of *B*. *burgdorferi* on rodent survival versus its effect on the recapture rate. Thus whether *B*. *burgdorferi* reduces the survival of *P*. *leucopus* has not been definitively resolved.

The purpose of the present study was to test whether *B*. *burgdorferi* infection and *I*. *scapularis* tick burden influence the survival and recapture rates of *P*. *leucopus*. To test this hypothesis, we analyzed the data from a published field study by Dolan et al. [38] where the authors used acaracides to reduce the tick burden and the proportion of *B*. *burgdorferi*-infected *P*. *leucopus* mice. This study was useful for three reasons: (1) four years of field data on a natural host-tick-pathogen interaction, (2) CMR data on *P*. *leucopus* allowed us to estimate a set of epidemiological parameters of interest (host survival and encounter rates, pathogen virulence, and infection rates), and (3) the acaracide treatment was highly effective and therefore provided a more powerful test of our hypothesis of interest. The capture effort in the present study (2181 captures) is ten-fold larger than the field study by Hofmeister *et al*. [32] (202 captures). Our study therefore provides the strongest test to date of whether *B*. *burgdorferi* influences the survival of its rodent host under natural conditions.

## Materials and Methods

The institutional animal care and use committee (IACUC) of the Centers for Disease Control and Prevention, Division of Vector-Borne Infectious Diseases approved this study. The study was carried out on private properties and all owners gave permission to conduct the study on their properties.

### Acaricide treatments

The field methods are covered in detail in Dolan et al. [38]. In brief, the purpose of their study was to reduce the burden of immature *I*. *scapularis* ticks on *P*. *leucopus* using rodent-targeted acaracides (fipronil and deltamethrin). The study was conducted on private properties on Mason’s Island (41.334923, -71.968228), near Mystic, Connecticut from 1999 to 2002. There was one control area, located in the undeveloped center of the island (41.333707, -71.967638), and there were three areas treated with acaracides: (1) Nauyaug Point on the southern tip of the island (41.321928, -71.969097), (2) Mallard Road on the northern part of the island (41.335769, -71.967165), and (3) the New Area on the eastern and central part of the island (41.332112, -71.963711). Nauyaug Point and Mallard Road were treated with fipronil (or deltamethrin) and sampled from 1999 to 2002 whereas the New Area was treated with fipronil and sampled from 2000 to 2002. In the three treated areas, rodents were targeted using acaricide-treated bait boxes but no such bait boxes were distributed in the control site. Thus any potential differences in rodent survival between the treated areas and the control area may be due to differences in parasite levels, differences in habitat quality (e.g. food levels), or both. In addition, there were substantial pre-existing differences in tick burden between the areas before the acaricide treatment. For example, in May 1999, the mean tick burden in the control area was almost 15 times higher than Nauyaug Point [38]. Differences among areas therefore include intrinsic differences in tick density in addition to the acaricide treatments. Finally, the inclusion of only one control site limits the generality of the results in the present study.

### Capture-mark-recapture of *P*. *leucopus* mice

For the CMR component of the study, mice were live-trapped using Sherman mousetraps on five trapping occasions for each year (May, June, July, August and September) in all four areas. Capture effort was constant over time and among areas. For each mouse, surveyors determined its sex, developmental stage (juvenile, subadult, adult), body weight, and tick burden. Tick burden was determined for each mouse without distinguishing between nymphal or larval ticks. We assumed that nymphs dominated the monthly tick burdens of May, June, and July whereas larvae dominated the monthly tick burdens of August and September. This assumption is supported by what is known about tick phenology in the region [38] and also by the monthly tick burden data itself, which generally showed two distinct peaks in May and August corresponding to the emergence of nymphal and larval ticks, respectively (see results). Surveyors also took an ear biopsy, which was subsequently cultured in BSK medium to determine whether the mouse was infected with *B*. *burgdorferi*. Previous studies have shown that ear biopsies are an efficient and sensitive method for establishing infection status in rodents [39, 40]. There were 115 mice for which *B*. *burgdorferi* infection status was not determined. Most of these individuals were from the sampling occasion on September 2000, when mice were captured but not checked for *B*. *burgdorferi* infection. These mice will be dealt with differently depending on the CMR statistical approach (see below). Surveyors captured numerous mice with ripped ears suggesting that there was considerable tag loss in this study (13.4% = 113/845). Studies with tag loss underestimate survival because a mouse that has lost its ear tag gives the false impression that it has left the study (by death or immigration). Importantly, there was no bias in tag loss between *B*. *burgdorferi*-infected (13.9% = 82/589) and uninfected mice (12.1% = 31/256).

## Statistical Methods

### Effect of acaricide treatment on tick burden and *B*. *burgdorferi* prevalence in *P*. *leucopus* mice

Dolan et al. [38] presented the data for the first three years (1999 to 2001) and for three of the four areas (control, Nauyaug Point, New Area). We re-analysed the data using generalized linear models (GLM) with binomial errors for the proportion of *B*. *burgdorferi*-infected mice and we used GLM models with negative binomial errors to re-analyze the tick burden data (see S1 File). The purpose of these analyses was to confirm that there were differences in the proportion of *B*. *burgdorferi*-infected mice and tick burden among areas, months, and years.

### Multistate-CMR models and parameters

**Fig 1:**
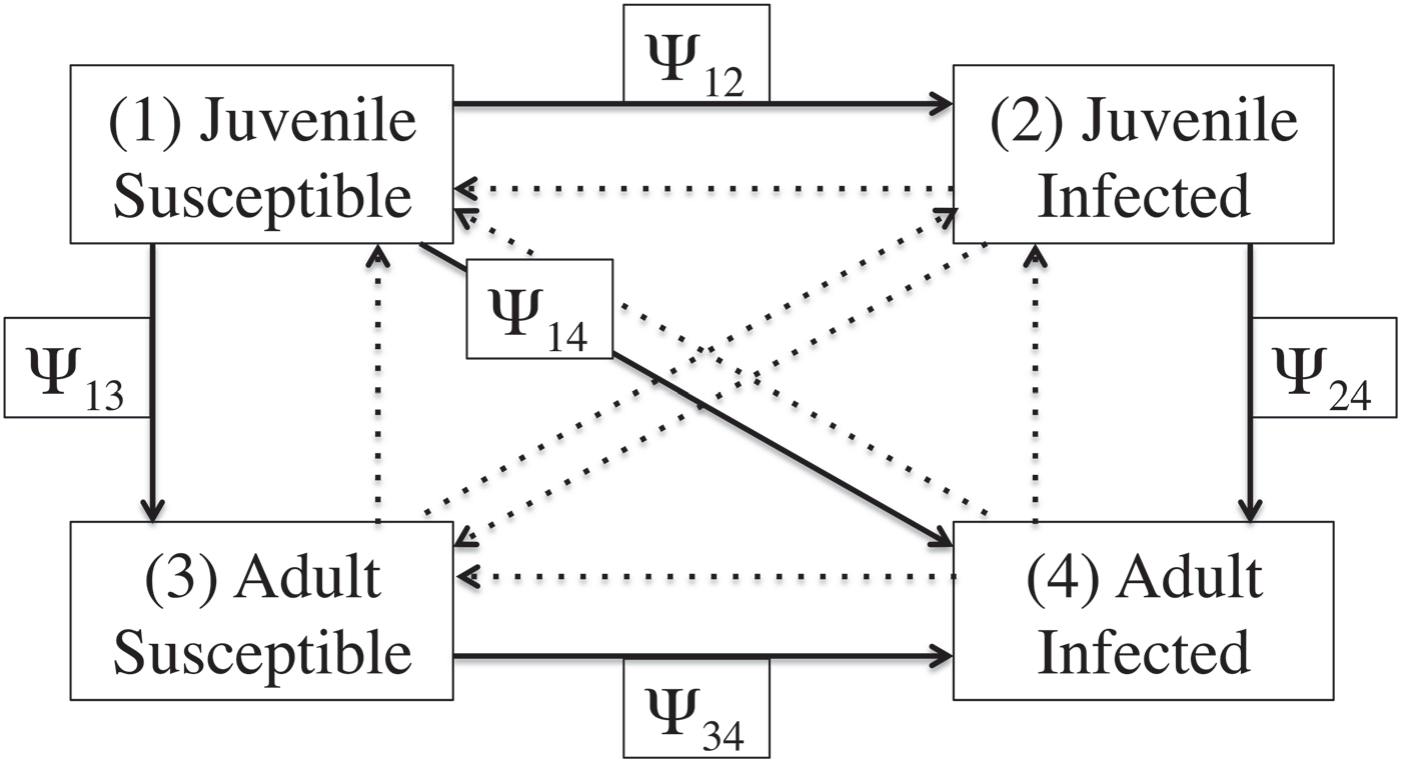
The transitions of the multistate model. The multistate model contained four states for the *Peromyscus leucopus* mice: (1) susceptible juveniles, (2) *Borrelia burgdorferi*-infected juveniles, (3) susceptible adults, and (4) *B*. *burgdorferi*-infected adults. The solid arrows show the five possible transitions in this system. The possible transitions are labeled, for example, the transition rate ψ_14_ is the probability that a susceptible juvenile (stage 1) will transition into an infected adult (stage 4) by the next capture occasion. The transition probabilities measure the instantaneous probability that an individual will change its state after surviving the time interval.

We used a multi-state model that allowed mice to transition among four distinct states: (1) susceptible juveniles, (2) *B*. *burgdorferi*-infected juveniles, (3) susceptible adults, and (4) *B*. *burgdorferi*-infected adults (Fig. 1). This model allowed us to test whether *B*. *burgdorferi* infection and developmental state affect mouse survival rates (ϕ) and recapture rates (p). This multi-state model also allowed us to estimate the transition probabilities (ψ) between states such as the probability that a juvenile will develop into an adult (α) or the probability that a mouse will acquire the infection (β) (Fig. 1). Thus multi-state CMR models simultaneously model three types of parameters: survival rates, recapture rates, and transition rates (such as development, infection, and recovery rates).

To simplify the modeling and to capture the relevant biology of the system, we only considered five of the twelve transition rates (Table 1). Adults cannot develop into juveniles and the transitions from adults to juveniles were therefore set to zero (Table 1). Wild rodents are infected for life following infection with *Borrelia* pathogens [25, 41] and we therefore set the recovery rate (transition from the infected to the uninfected state) to zero (Table 1). The transition of susceptible juveniles to infected adults is possible and will hereafter be referred to as the ‘delta’ transition (δ). The delta transition is the product of the developmental rate (α) and the infection rate (β) but MARK is currently unable to decompose such two-step transitions into the constituent one-step transitions. Thus we modeled five multi-state CMR parameters for the *P*. *leucopus* mice: survival rate (ϕ), recapture rate (p), infection rate (β), developmental rate (α), and the delta transition (δ).

**Table 1:** Summary of the transition rates.

Transition	Description
ψ_12_ = β_j_	Susceptible juvenile acquires *B*. *burgdorferi* infection
ψ_13_ = α_s_	Susceptible juvenile develops into a susceptible adult
ψ_14_ = δ	Susceptible juvenile develops into an infected adult
ψ_21_ = 0	Infected juvenile recovers from *B*. *burgdorferi* infection
ψ_23_ = 0	Infected juvenile develops into a susceptible adult
ψ_24_ = α_i_	Infected juvenile develops into an infected adult
ψ_31_ = 0	Susceptible adult regresses into a susceptible juvenile
ψ_32_ = 0	Susceptible adult regresses into an infected juvenile
ψ_34_ = β_a_	Susceptible adult acquires *B*. *burgdorferi* infection
ψ_41_ = 0	Infected adult regresses into a susceptible juvenile
ψ_42_ = 0	Infected adult regresses into an infected juvenile
ψ_43_ = 0	Infected adult recovers from *B*. *burgdorferi* infection

The multistate model contained four states for the *Peromyscus leucopus* mice: (1) susceptible juveniles, (2) *Borrelia burgdorferi*-infected juveniles, (3) susceptible adults, and (4) *B*. *burgdorferi*-infected adults. The type of transition is indicated by the subscripts on the transition rate parameter (ψ). For example, ψ_12_ indicates that a susceptible juvenile (state 1) transitioned into an infected juvenile (state 2). We we modeled five of the twelve theoretical transitions; the other seven transitions, which are not biologically possible, were set to zero. For example, adult mice cannot develop into juvenile mice. The transition from the uninfected to the infected state was symbolized with the infection rate parameter (β). The transition from the juvenile to the adult state was symbolized with the developmental rate parameter (α). The two-step transition from the uninfected juvenile to the infected adult state was symbolized with the delta rate parameter (δ). The subscripts of the infection rates (β) and developmental rates (α) refer to the identity of the other state. Thus, β_j_ and β_a_ refer to the infection rates of juveniles and adults whereas α_s_ and α_i_ refer to the developmental rates of susceptible and infected individuals, respectively.

In the results, the survival rate refers to the probability that a mouse will survive a period of 30 days and will therefore be referred to as the monthly survival rate. The recapture rate refers to the probability of encountering a marked mouse during a given sampling occasion. The developmental rate refers to the probability that a mouse in the juvenile state at time *i* is in the adult state at time *i* + 1, given that the animal is alive at time *i* + 1. The infection rate refers to the probability that a mouse in the uninfected state at time *i* is in the infected state at time *i* + 1, given that the animal is alive at time *i* + 1. The delta rate refers to the probability that a mouse in the uninfected juvenile state at time *i* is in the infected adult state at time *i* + 1, given that the animal is alive at time *i* + 1. When not otherwise specified, all parameter estimates refer to a susceptible adult female mouse captured in the control area in May 2000. The multi-state CMR parameters are all probabilities and therefore do not have units.

### Fixed factors and population-level covariates

We modeled the survival, recapture, and transition rates as a function of developmental state (2 levels: juveniles, adults; due to small sample sizes, juveniles and subadults were combined into a single developmental state), *B*. *burgdorferi* infection state (2 levels: susceptible, infected), sex (2 levels: female, male), area (4 levels: control area, Mallard Road, Nauyaug Point, New Area), year (4 levels: 1999, 2000, 2001, 2002), and month (5 levels: May, June, July, August, September). In addition, we used population-level covariates such as the monthly or yearly *B*. *burgdorferi* prevalence and the monthly or yearly tick burden in the mouse population for each of the four areas separately. Thus mouse survival was modeled as a function of *B*. *burgdorferi* infection in two distinct ways: (1) as a state variable where the individual is susceptible or infected, and (2) as a population-level covariate (the monthly or yearly area-specific proportion of *B*. *burgdorferi*-infected mice). We explain the logic for these two approaches in the section titled, “Acute mortality and undetected infections”.

### Population-level covariates—prevalence of *B*. *burgdorferi*

The proportion of mice infected with *B*. *burgdorferi* infection was used as a population-level, linear covariate. The area-specific proportion of *B*. *burgdorferi*-infected mice was calculated for two different time scales: (1) the monthly area-specific prevalence and (2) the yearly area-specific prevalence. For the monthly area-specific prevalence, we modeled survival as a function of the estimates at the start (and not the end) of the time interval. One disadvantage of this approach was that survival over the 8-month sampling hiatus (between September and May) depended on the September prevalence of *B*. *burgdorferi*. For the yearly area-specific prevalence, we modeled mouse survival as a function of the proportion of *B*. *burgdorferi*-infected mice for the entire year.

### Population-level covariates—tick burden

Tick burden was also modeled as a monthly or an annual area-specific population-level covariate. We modeled tick burden this way because we felt that the large seasonal fluctuations in tick burden would have the most important implications for the survival cost of tick ectoparasites on mice and for the infection risk of *B*. *burgdorferi*. A limitation of modeling tick burden as a population-level covariate is that it assumes that the tick burden is equal for all mice. In reality, ticks are often highly aggregated on a fraction of the mouse population [42–44]. Modeling tick burden as an individual covariate is currently not possible for multistate models in the MARK software. In summary, tick burden was modeled as a population-level covariate but not as a state variable.

### Population-level covariates—tick-induced blood loss

**Fig 2:**
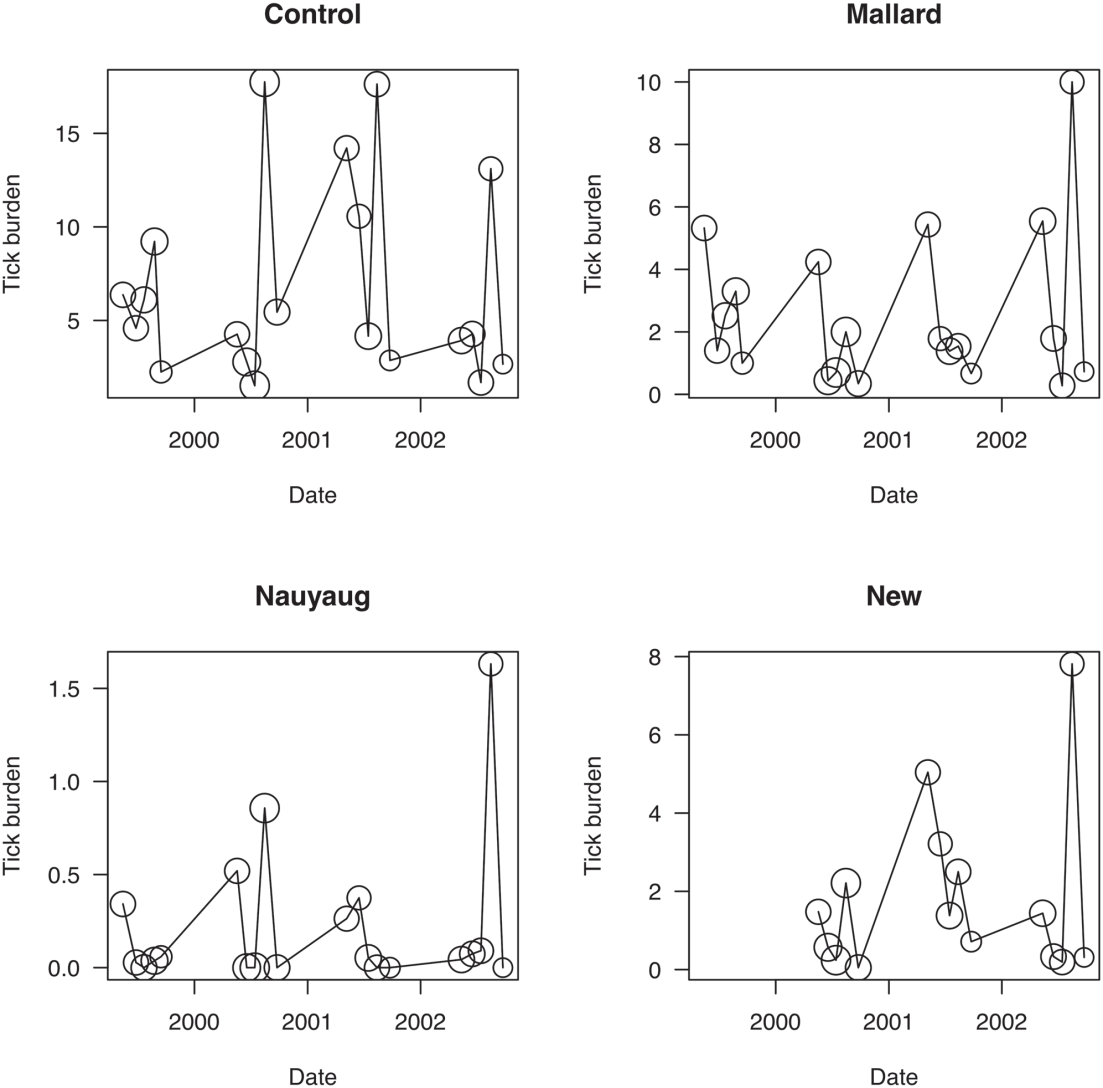
Seasonal and annual variation in the tick burden on mice. Average monthly burden of immature *Ixodes scapularis* ticks (larvae and nymphs) on *Peromyscus leucopus* mice showed a seasonal pattern over the four years of the study (1999, 2000, 2001, and 2002) for each of the four areas (Control Area, Mallard Road, Nauyaug Point, New Area). Tick burden has units of number of ticks per mouse. There are five sampling months within each year (May, June, July, August, September). The size of the circle is proportional to the number of mice on which the average is based (range: 1 to 64). The properties in the New Area were not sampled in 1999.

To better model the survival costs caused by tick-induced blood loss, we took into account that nymphs take much larger blood meals than larvae. The mean size of a tick blood meal was based on the data of Balashov (1972) for *Ixodes persulcatus*: 2.62 μl for a larva and 15.86 μl for a nymph. We calculated the monthly tick-induced blood loss under the assumption that the tick burden in May, June, and July consists of nymphs whereas the tick burden in August and September consists of larvae (Fig. 2).

### Population-level covariates—burden of infected nymphs

The rate at which mice acquire the *B*. *burgdorferi* infection depends on the density of infected nymphs (DIN) [45, 46]. The mean annual tick burden is a poor estimate of the DIN because this variable is biased high by larvae. A better estimate of the DIN would calculate the mean annual nymph burden over the months of May, June, and July and exclude the larval tick months of August and September. To best model the infection rate, we calculated two additional population-level tick burden variables, the annual burden of infected nymphs indices 1 and 2 (BIN_1_ and BIN_2_). BIN_1_ corrects the annual nymph burden for the proportion of larvae that acquired the spirochete from infected *P*. *leucopus* mice the previous year and BIN_2_ corrects BIN_1_ for the proportion of larvae that feed on non-competent reservoir hosts (see S2 File).

### Acute mortality and undetected infections

Many diseases are characterized by an initial acute phase where mortality may be substantially higher than in the subsequent chronic phase. If the acute phase of the disease is short relative to the time interval between captures, then individuals might acquire the infection and die between sampling occasions. These individuals have capture histories terminating in the uninfected state. Failure to detect acute infections will underestimate survival of uninfected hosts, overestimate survival of infected hosts, and underestimate infection rates. If the acute phase of the disease removes weak individuals with intrinsically low survival rates, the chronically infected population may have higher survival than the susceptible population, which has not been exposed to this pathogen-mediated viability selection. Other investigators have previously discussed this problem of missing acute infections [7, 9, 14]. One obvious solution is to sample more frequently but this is not always feasible. Telfer et al. (2002) reasoned that, “In such cases, looking for correlations between pathogen prevalence and survival rates at the population level may prove more informative. If a large number of infected individuals are dying [before detecting their infection status], average survival rates should be lower when [pathogen] prevalence is high.” [14]. Similarly, Lachish et al. (2011) reasoned that, “if purportedly uninfected hosts have lower survival rates in high-prevalence areas, where the force of infection is high, then this would indicate that acute infections carry a fitness cost for hosts (because [hosts] in high-prevalence areas would be more likely to have acquired infection and died soon after infection without this transition appearing in our data set).” [9]. Thus these approaches test whether population-wide measures of pathogen prevalence influence host survival. We therefore modeled mouse survival as a function of *B*. *burgdorferi* prevalence (a population-level covariate) to account for any acute phase infection effects.

### CMR data—captures, states and transitions

The CMR data set contained 21 sampling occasions from May 1999 to May 2003 and thus 20 time intervals over 4 years. The data set contained 1567 unique mice, 2181 captures and 614 recaptures (2181 captures/1567 individuals = 1.39 captures/individual). As there were only 71 juveniles and 121 sub-adults, we combined these two categories into a single ‘juvenile’ state. Our data set contained 169 susceptible juveniles, 23 infected juveniles, 836 susceptible adults, 1038 infected adults, and 115 adults for which the infection state was not determined. We observed 148 state transitions: 32 juvenile to adult transitions (25 susceptible and 7 infected individuals), 105 uninfected to infected transitions (1 juvenile and 104 adult individuals), and 11 delta transitions where a susceptible juvenile developped into an infected adult. We observed 13 apparent recoveries where an infected adult transitioned into an uninfected adult. We believe that these 13 apparent recoveries represent undetected infections (false negatives) and not true recoveries. We therefore recoded these 13 adult mice as having chronic infections (i.e. infected → susceptible was recoded as infected → infected).

### Handling missing data

There were 115 adults from the September 2000 capture session for which the infection state was not determined. Under the assumption of no recovery, we recoded 30 of the 115 adults with unknown infection states as being infected (i.e. infected → unknown was recoded as infected → infected). This left 85 adults for which the infection state remained unknown. We did not want to remove these individuals from the analysis because this would bias the survival and recapture rates. For these 85 adults, we conducted one analysis where the the unknown states were coded as susceptible (910 susceptible adults, 1080 infected adults, and 94 adult infection events). We then conducted a second analysis where the unknown states were coded as infected (825 susceptible adults, 1164 infected adults, and 104 adult infection events). There are more sophisticated approaches to dealing with state uncertainty [10, 15, 47], but our approach was adequate because the fraction of unknown states was low (85/2181 captures = 3.9%) and because the conclusions were similar for the two data sets (see results).

For the New Area, which was not sampled in 1999, the five missing sampling occasions were coded with missing values (dots), which allowed the comparison of groups where sampling started on different occasions. There were 11 individuals that died in the trap and these individuals were removed from the study by coding their capture history frequency with-1 in the MARK input file.

### *A priori* expectations for the multi-state CMR models

We modeled five multi-state CMR parameters: survival rate (ϕ), recapture rate (p), infection rate (β), developmental rate (α), and the delta transition (δ). We had strong *a priori* expectations that survival, recapture, and infection rates would depend on area and month. In addition, we expected that the infection rate would depend on stage (adults are more likely to become infected than juveniles) and that the developmental rate would depend on infection status (*B*. *burgdorferi*-infected juveniles develop ‘faster’ because they are older than susceptible juveniles). Thus our starting model was: ϕ(area+month) p(area+month) β(stage+area+month) α(Bb) δ(.). We first tested whether the starting model could be simplified by deleting each explanatory factor from each parameter. We then sequentially modeled the delta transition (δ), the developmental rate (α), the infection rate (β), the recapture rate (p), and finally the survival rate (ϕ). Each time we found a better model for a given parameter, we updated the starting model for the next parameter in the sequence. In general, our goal was to compare models that contained generic variables for space and time (the factors area and month) with models that contained the Lyme disease variables (*B*. *burgdorferi* infection status, monthly or yearly estimates of *B*. *burgdorferi* prevalence, monthly or yearly estimates of tick burden and tick burden-induced blood loss). We tested some two-way interactions but did not test any three-way interactions because the data was too sparse.

### Model selection approach using AIC

We used a model selection approach based on the Akaike Information Criterion corrected for small sample size (AICc) [4, 48]. Models were ranked according to their AICc values. The model with the lowest AICc value is considered to be the best model because it minimizes both the unexplained variation (residual deviance) and the precision (standard error) of the parameter estimates. The differences in AICc value from the top model were used to assign weight or support to all the models in the candidate set. These values of weight or support were used to determine the importance of the explanatory factors for the different CMR parameters. Burnham and Anderson [48] strongly recommend against mixing information-theoretic approaches (used in the present study) with classical null hypothesis testing and we therefore do not report p-values in the results.

The AICc value of a model depends on its residual deviance and the number of parameters. The software MARK does not count parameters that it fails to estimate thereby underestimating the true AICc value of the model. To avoid this problem, we always calculated the AICc value for each model using the expected number of parameters rather than the number of parameters counted by MARK. The model-averaged parameter estimates were calculated by weighting the CMR parameter estimates of each model in the candidate model set by its AICc model weight. These model-averaged CMR parameter estimates contain the model uncertainty that is inherent when considering multiple alternative models.

### Goodness of fit testing

We used the program U-CARE to test whether our CMR data met the assumptions of the multi-state CMR analysis [49]. We tested whether the JollyMove (JMV) model fit the data for each of the four areas (sexes were combined for each area). The goodness of fit test found that the data fit the JMV model for each of the four areas (p-values for ranged between 0.697 and 0.999).

## Results

### *I*. *scapularis* burden and *B*. *burgdorferi* infection prevalence

**Fig 3:**
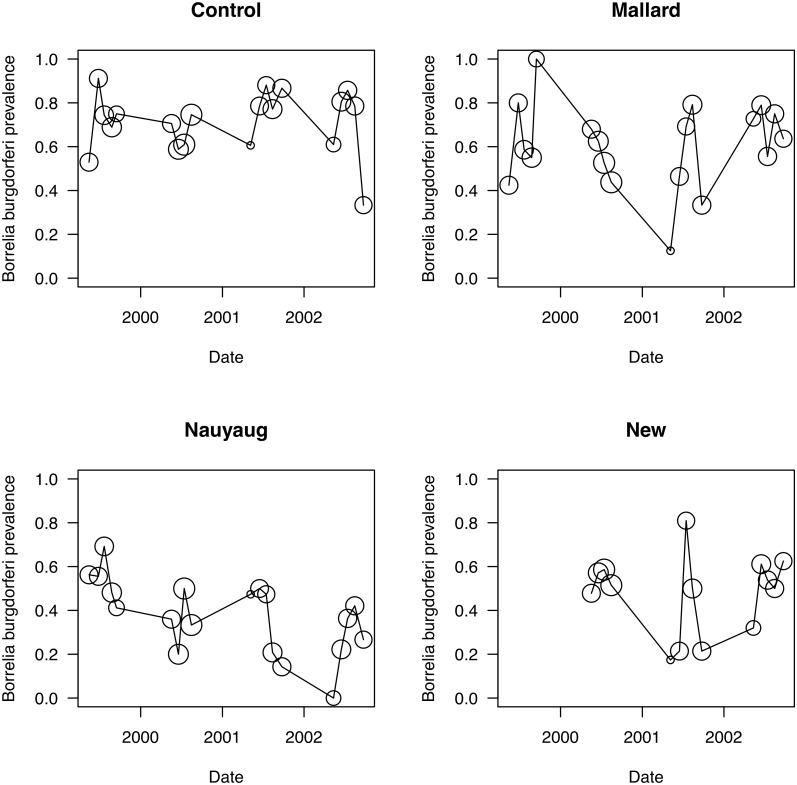
Seasonal and annual variation in *Borrelia burgdorferi* infection of mice. Monthly prevalence of *Borrelia burgdorferi*-infected *Peromyscus leucopus* mice varied over the four years of the study (1999, 2000, 2001, and 2002) for each of the four areas (Control Area, Mallard Road, Nauyaug Point, New Area). The prevalence is a proportion and therefore does not have units. There are five sampling months within each year (May, June, July, August, September). The size of the circle is proportional to the number of mice on which the prevalence is based (range: 1 to 64). The properties in the New Area were not sampled in 1999. There is no estimate for September 2000 because the mice were not tested for *B*. *burgdorferi* infection.

The mean tick burden and the proportion of *B*. *burgdorferi*-infected mice varied seasonally and among the four areas (Figs. 2 and 3). The mean tick burden (± standard error) was highest in the control area (7.1 ± 0.46 ticks/mouse; n = 728), intermediate in Mallard Road (2.5 ± 0.29; n = 373) and the New Area (1.7 ± 0.27; n = 323), and lowest in Nauyaug Point (0.2 ± 0.05; n = 437; Fig. 2). Similarly the proportion of *B*. *burgdorferi*-infected mice was highest in the control area (0.716), intermediate in Mallard Road (0.573) and the New Area (0.484), and lowest in Nauyaug Point (0.393; Fig. 3). In all four years of the study in the control area, the mean tick burden exhibited seasonal fluctuations that are consistent with what is known about the population dynamics of *I*. *scapularis* in the region; a small peak of nymphal ticks in May preceded a much larger peak of larval ticks in August (Fig. 2). In the acaricide-treated areas by contrast, the nymphal peak was larger than the larval peak in 6 out of 11 cases because the acaricide treatment suppressed the larval burden on the mice. The proportion of *B*. *burgdorferi*-infected mice was higher in the control area than in the acaricide-treated areas and appeared to decline over time in Nauyaug Point (Fig. 3). Juvenile and sub-adult mice had lower tick burdens and *B*. *burgdorferi* infection levels than adult mice (Table 2). Adult female mice had lower tick burdens and *B*. *burgdorferi* infection levels than adult male mice (Table 2).

**Table 2:** Body weight, tick burden, and prevalence of *Borrelia* infection for the six combinations of sex and stage.

Sex	Stage	n	Weight ± se	Ticks ± se	*Borrelia* ± se
male	juv	62	12.8 ± 0.37	3.7 ± 2.82	0.059 ± 0.0269
male	sub	92	16.6 ± 0.18	1.3 ± 0.32	0.159 ± 0.0380
male	adult	995	22.4 ± 0.12	4.5 ± 0.28	0.525 ± 0.0153
female	juv	47	12.6 ± 0.49	1.3 ± 0.52	0.078 ± 0.0351
female	sub	84	16.5 ± 0.28	1.7 ± 0.55	0.145 ± 0.0358
female	adult	826	22.8 ± 0.14	2.3 ± 0.25	0.423 ± 0.0167

Summary data (means ± standard error) for body weight (grams), tick burden (number of ticks per mouse) and the proportion of *Peromyscus leucopus* mice infected with *Borrelia burgdorferi* are shown for the six combinations of sex and stage (juvenile, subadult, and adult). The sample size (n) was calculated across all years and areas of the study.

### Multi-state CMR models

The starting model (model A00 in Table 3), ϕ(area+month) p(area+month) β(stage+area+month) α(Bb) δ(.), was a much better fit to the data than the JollyMove (JMV) model (model G01 in Table 3), which we used to test the assumptions of the multi-state CMR analysis. Model simplification (models A01 to A06 in Table 3) found a better starting model (model A01 in Table 3), ϕ(area+month) p(month) β(stage+area+month) α(Bb) δ(.), where the recapture rate depended only on month.

**Table 3:** Multistate capture-mark-recapture (CMR) models of the *Peromyscus leucopus* mice.

Model ID	Model Structure	Par	Parameter Model Structure	AICc	Δ AICc	Weights	N	Deviance
**E01**	**p(Bb+x+Bb:x+m) β(s+a+m+y) α(Bb) δ(.)**	**φ**	**a+m**	**3791.5**	**0.0**	**0.490**	**31**	**1955.0**
F01	p(Bb+x+Bb:x+m) β(s+a+m+y) α(Bb) δ(.)	φ	Bb+x+Bb:x+a+m	3794.2	2.7	0.129	34	1951.4
F02	p(Bb+x+Bb:x+m) β(s+a+m+y) α(Bb) δ(.)	φ	a+m+Prev+a:Prev	3795.0	3.5	0.085	35	1950.2
F03	p(Bb+x+Bb:x+m) β(s+a+m+y) α(Bb) δ(.)	φ	a+m+Blood+a:Blood	3795.4	3.9	0.069	35	1950.6
F04	p(Bb+x+Bb:x+m) β(s+a+m+y) α(Bb) δ(.)	φ	a+m+Tick.m+a:Tick.m	3795.9	4.4	0.054	35	1951.1
F05	p(Bb+x+Bb:x+m) β(s+a+m+y) α(Bb) δ(.)	φ	s+Bb+s:Bb+a+m	3796.0	4.5	0.051	34	1953.3
F06	p(Bb+x+Bb:x+m) β(s+a+m+y) α(Bb) δ(.)	φ	s+x+s:x+a+m	3796.2	4.7	0.048	34	1953.4
F07	p(Bb+x+Bb:x+m) β(s+a+m+y) α(Bb) δ(.)	φ	s*Bb*x-s:Bb:x+a+m	3799.3	7.8	0.010	37	1950.3
F08	p(Bb+x+Bb:x+m) β(s+a+m+y) α(Bb) δ(.)	φ	s*Bb*x+a+m	3800.5	8.9	0.006	38	1949.4
E01	φ(a+m) β(s+a+m+y) α(Bb) δ(.)	p	Bb+x+Bb:x+m	3791.5	0.0	0.490	31	1955.0
E02	φ(a+m) β(s+a+m+y) α(Bb) δ(.)	p	s+Bb+x+s:Bb+s:x+Bb:x+m	3797.2	5.7	0.028	34	1954.5
E03	φ(a+m) β(s+a+m+y) α(Bb) δ(.)	p	s+Bb+x+s:Bb+s:x+Bb:x+s:Bb:x+m	3798.1	6.6	0.018	35	1953.3
E04	φ(a+m) β(s+a+m+y) α(Bb) δ(.)	p	s+Bb+s:Bb+m	3801.2	9.7	0.004	31	1964.6
**D01**	**φ(a+m) β(s+a+m+y) α(Bb) δ(.)**	**p**	**m**	**3802.6**	**11.0**	**0.002**	**28**	**1972.2**
E05	φ(a+m) β(s+a+m+y) α(Bb) δ(.)	p	s+x+s:x+m	3805.6	14.0	0.000	31	1969.0
D01	φ(a+m) p(m) α(Bb) δ(.)	β	s+a+m+y	3802.6	11.0	0.002	28	1972.2
D02	φ(a+m) p(m) α(Bb) δ(.)	β	s+x+s:x+BIN+s:BIN+m+y	3802.6	11.1	0.002	29	1970.2
D03	φ(a+m) p(m) α(Bb) δ(.)	β	s+BIN+s:BIN+m	3804.8	13.3	0.001	24	1982.7
D04	φ(a+m) p(m) α(Bb) δ(.)	β	s+nymph+s:nymph+m	3808.1	16.6	0.000	24	1985.9
D05	φ(a+m) p(m) α(Bb) δ(.)	β	s+x+s:x+a+m	3813.4	21.9	0.000	27	1985.1
D06	φ(a+m) p(m) α(Bb) δ(.)	β	s+a+m+y+a:y	3815.1	23.6	0.000	37	1966.2
**A01**	**φ(a+m) p(m) α(Bb) δ(.)**	**β**	**s+a+m**	**3815.3**	**23.8**	**0.000**	**25**	**1991.1**
D07	φ(a+m) p(m) α(Bb) δ(.)	β	s+Tick.y+s:Tick.y+m	3817.8	26.3	0.000	24	1995.7
D08	φ(a+m) p(m) α(Bb) δ(.)	β	s+a+m+a:m	3820.2	28.7	0.000	37	1971.3
D09	φ(a+m) p(m) α(Bb) δ(.)	β	s+a+y+a:y	3826.5	35.0	0.000	33	1985.8
D10	φ(a+m) p(m) α(Bb) δ(.)	β	s+Tick.m+s:Tick.m+m	3832.9	41.3	0.000	24	2010.7
A01	φ(a+m) p(m) β(s+a+m) δ(.)	α	Bb	3815.3	23.8	0.000	25	1991.1
C01	φ(a+m) p(m) β(s+a+m) δ(.)	α	Bb+x	3817.0	25.5	0.000	26	1990.7
C02	φ(a+m) p(m) β(s+a+m) δ(.)	α	Bb+x+Bb:x	3819.4	27.9	0.000	27	1991.1
C03	φ(a+m) p(m) β(s+a+m) δ(.)	α	x	3820.4	28.8	0.000	25	1996.2
C04	φ(a+m) p(m) β(s+a+m) δ(.)	α	Bb+BIN+Bb:BIN	3822.2	30.7	0.000	27	1993.9
C05	φ(a+m) p(m) β(s+a+m) δ(.)	α	Bb+Blood+Bb:Blood	3822.9	31.4	0.000	27	1994.6
C06	φ(a+m) p(m) β(s+a+m) δ(.)	α	Bb+Tick.y+Bb:Tick.y	3824.1	32.6	0.000	27	1995.8
A01	φ(a+m) p(m) β(s+a+m) α(Bb)	δ	.	3815.3	23.8	0.000	25	1991.1
B01	φ(a+m) p(m) β(s+a+m) α(Bb)	δ	x	3816.4	24.9	0.000	26	1990.1
B02	φ(a+m) p(m) β(s+a+m) α(Bb)	δ	m	3823.9	32.3	0.000	29	1991.4
B03	φ(a+m) p(m) β(s+a+m) α(Bb)	δ	a+x	3827.8	36.3	0.000	29	1995.4
B04	φ(a+m) p(m) β(s+a+m) α(Bb)	δ	a	3827.8	36.3	0.000	28	1997.4
A01	φ(a+m) p(m) β(s+a+m) α(Bb) δ(.)			3815.3	23.8	0.000	25	1991.1
A00	φ(a+m) p(a+m) β(s+a+m) α(Bb) δ(.)			3815.9	24.4	0.000	28	1985.5
A02	φ(a+m) p(a+m) β(s+a+m) α(.) δ(.)			3819.3	27.7	0.000	27	1991.0
A03	φ(a+m) p(a+m) β(s+a) α(Bb) δ(.)			3826.6	35.1	0.000	24	2004.5
A04	φ(m) p(a+m) β(s+a+m) α(Bb) δ(.)			3832.0	40.5	0.000	25	2007.8
A05	φ(a+m) p(a) β(s+a+m) α(Bb) δ(.)			3836.0	44.5	0.000	24	2013.9
A06	φ(a) p(a+m) β(s+a+m) α(Bb) δ(.)			3851.4	59.8	0.000	24	2029.2
A07	φ(a+m) p(a+m) β(s+m) α(Bb) δ(.)			3866.5	75.0	0.000	25	2042.3
G01	φ(time) p(time) α(.) β(time) δ(.)			3886.5	95.0	0.000	63	1982.9

Model selection results from the CMR model with four states: (1) susceptible juveniles, (2) *Borrelia burgdorferi*-infected juveniles, (3) susceptible adults, and (4) *B*. *burgdorferi*-infected adults. There are five CMR parameters: monthly survival rate (φ), recapture rate (p), infection rate (β), developmental rate (α), and delta transition rate (δ). Explanatory factors include: *B*. *burgdorferi* infection status (Bb), stage (s), sex (x), area (a), month (m), year (y), monthly area-specific prevalence of *B*. *burgdorferi* in the mouse population (Prev), monthly area-specific *Ixodes scapularis* tick burden in the mouse population (Tick.m), annual area-specific tick burden in the mouse population (Tick.y), annual area-specific tick-induced blood loss in the mouse population (Blood), annual area-specific nymphal burden in the mouse population (nymph), and the annual area-specific burden of infected nymphs (BIN). The structure of the starting model (A00) was based on a priori predictions. Models A01 to A07 were reduced versions of the starting model. The five parameters were modeled in the following sequence: δ transition (models B01 to B04), development (models C01 to C06), infection (models D01 to D10), recapture (models E01 to E05), and survival (models F01 to F08). We used U-CARE to test whether model G01 met the assumptions of the multi-state CMR analysis.

### Survival rate

**Fig 4:**
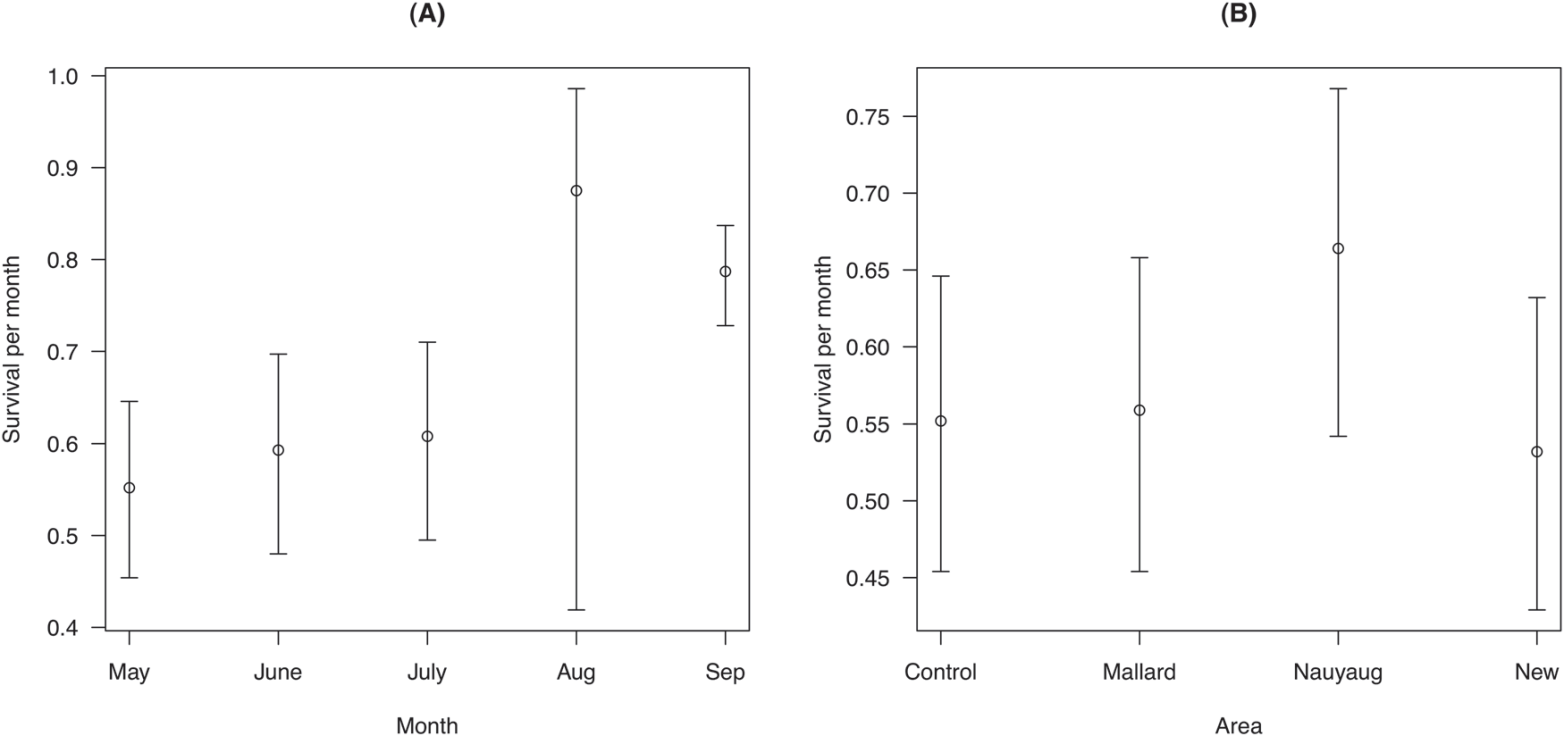
Effect of month and area on the mouse survival rate. Monthly survival rates for *Peromyscus leucopus* mice (A) increased over the course of the summer and (B) differed among the four areas. The survival rate is the probability that a mouse will survive a period of 30 days. The survival rates are shown for a reference mouse, which is an adult female susceptible mouse living in the control area in May 2000. Shown are the means and the 95% confidence limits. The parameter estimates include variation due to model uncertainty.

Models of the survival rate (models F01 to F08 in Table 3) found that this parameter was best modeled as a function of area and month (model E01 in Table 3). The model-averaged mean survival rate (± S. E.) for an adult female susceptible mouse in the control area in May 2000 was 0.552 ± 0.050. In what follows, this value will be used as the reference survival rate (ϕ_reference_). Survival rates were higher in August (0.875 ± 0.127) and September (0.787 ± 0.028) than the other three months: May (ϕ_reference_), June (0.593 ± 0.056), and July (0.608 ± 0.056; Fig. 4). Survival rates were also higher in Nauyaug Point (0.664 ± 0.059) than the other three areas: control area (ϕ_reference_), Mallard Road (0.559 ± 0.053), and the New Area (0.532 ± 0.053; Fig. 4). There was no support that mouse survival was influenced by *B*. *burgdorferi* infection status, the monthly prevalence of *B*. *burgdorferi*, the monthly tick burden, or the monthly tick-induced blood loss (models F01, F02, F04, and F03 in Table 3).

### Recapture rate

**Fig 5:**
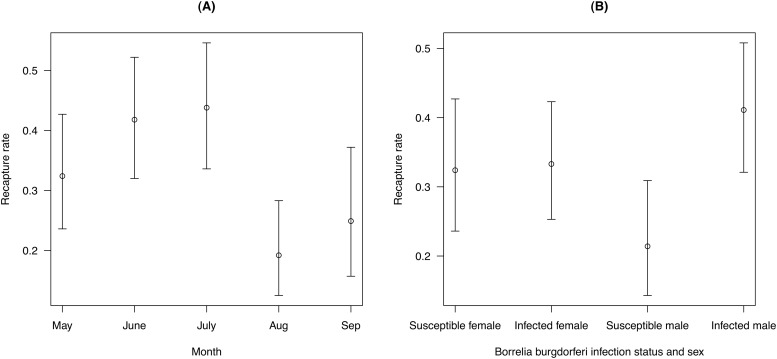
Effect of month, sex, and *Borrelia* infection on the mouse recapture rate. Recapture rates for (A) *Peromyscus leucopus* mice over the course of the summer and for (B) susceptible and infected adult female and male mice. The recapture rate is the probability of encountering a marked mouse during a given sampling occasion. The recaptures rates are shown for a reference mouse, which is an adult female susceptible mouse living in the control area in May 2000. Shown are the means and the 95% confidence limits. The parameter estimates include variation due to model uncertainty.

Models of the recapture rate (models E01 to E05 in Table 3) found that this parameter was best modeled as a function of *B*. *burgdorferi* infection, sex, the infection:sex interaction, and month (model E01 in Table 3). The model-averaged mean recapture rate (± S. E.) for an adult female susceptible mouse in the control area in May 2000 was 0.324 ± 0.049. In what follows, this value will be used as the reference recapture rate (p_reference_). The recapture rate of *B*. *burgdorferi*-infected males (0.411 ± 0.048) was almost twice as high as that of susceptible males (0.214 ± 0.042). In contrast, there was no difference in the recapture rate between infected (0.333 ± 0.044) and uninfected females (p_reference_; Fig. 5). Recapture rates were lower in August (0.192 ± 0.040) and September (0.249 ± 0.055) than in the other three months: May (p_reference_), June (0.418 ± 0.052), and July (0.438 ± 0.054; Fig. 5).

### Infection rate

**Fig 6:**
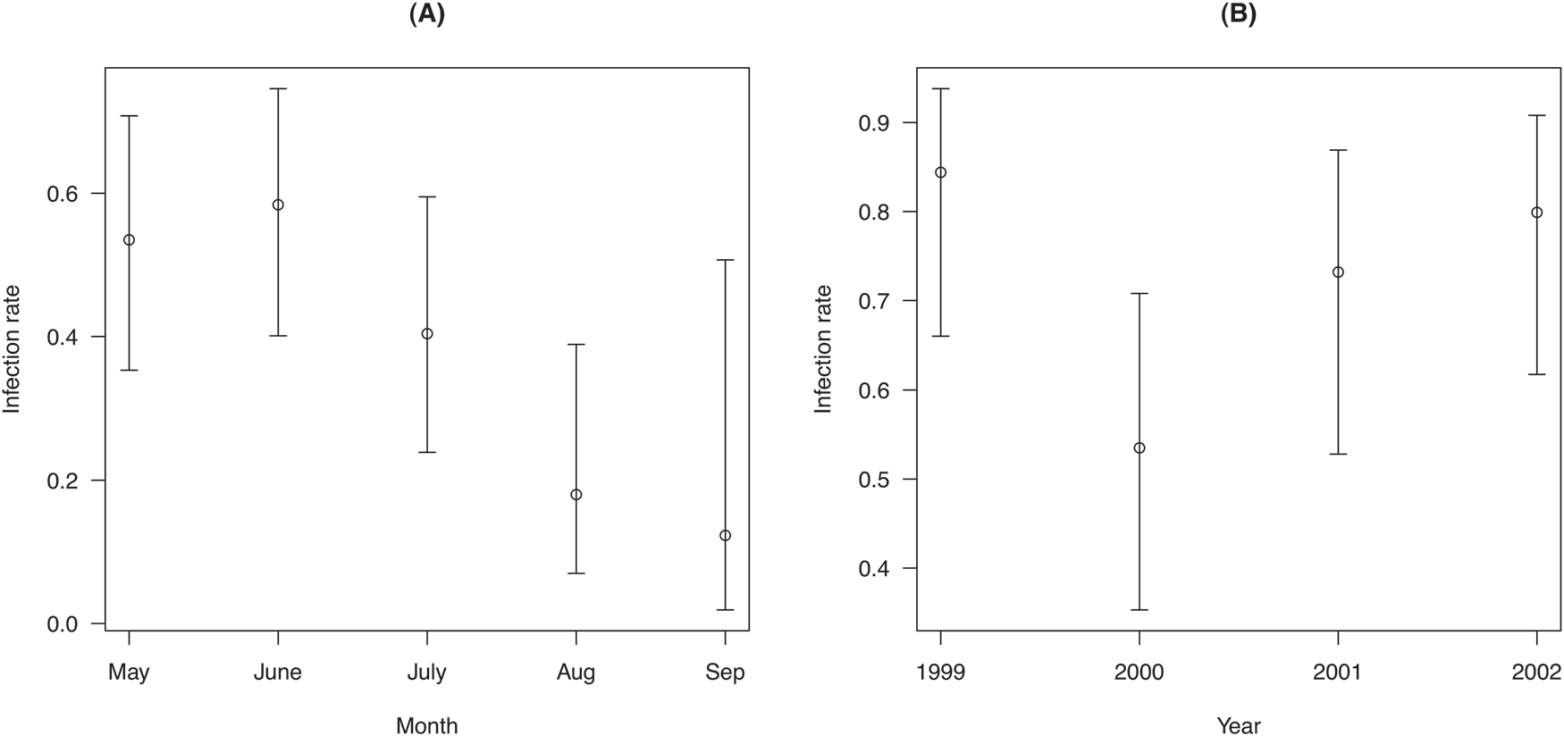
Effect of month and year on the infection rate. The infection rate of *Peromyscus leucopus* mice (A) decreased over the course of the summer and (B) differed among years of the study. The infection rate is the instantaneous probability that a mouse will acquire a *Borrelia burgdorferi* infection after surviving through a time interval of 30 days. The infection rates are shown for a reference mouse, which is an adult female susceptible mouse living in the control area in May 2000. Shown are the means and the 95% confidence limits. The parameter estimates include variation due to model uncertainty.

**Fig 7:**
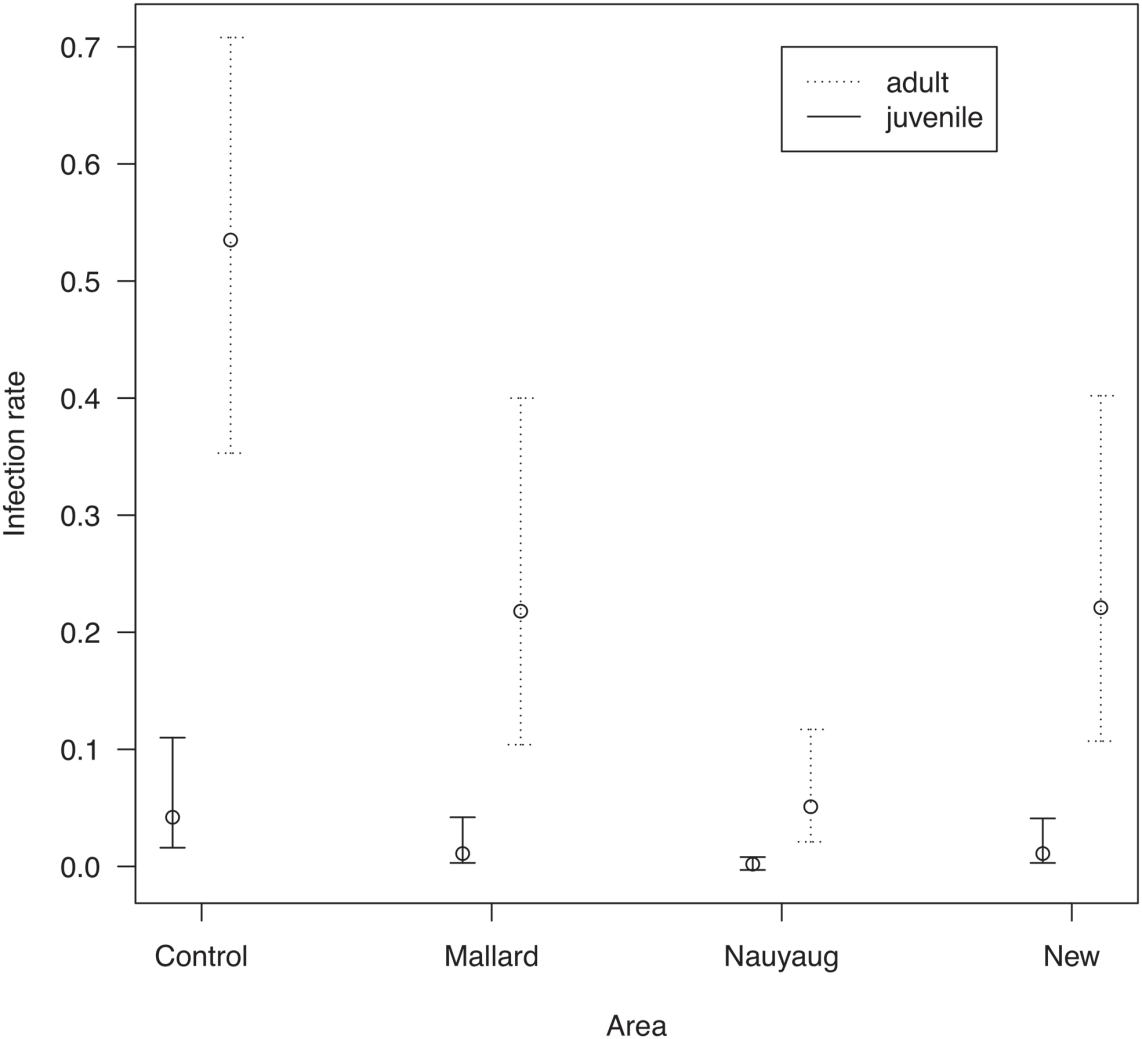
Effect of stage and area on the infection rate. The infection rate was higher for adult than juvenile *Peromyscus leucopus* mice and differed among the four areas. The infection rate is the instantaneous probability that a mouse will acquire a *Borrelia burgdorferi* infection after surviving through a time interval of 30 days. The infection rates are shown for a reference mouse, which is an adult female susceptible mouse living in the control area in May 2000. Shown are the means and the 95% confidence limits. The parameter estimates include variation due to model uncertainty.

Models of the infection rate (models D01 to D10 in Table 3) found that this parameter was best modeled as a function of stage, area, month and year (model D01 in Table 3). The model-averaged mean infection rate (± S. E.) for an adult female susceptible mouse in the control area in May 2000 was 0.535 ± 0.095. In what follows, this value will be used as the reference infection rate (β_reference_). The *B*. *burgdorferi* infection rate decreased over the course of the summer; it was almost five times higher in June (0.584 ± 0.092) than in September (0.123 ± 0.110; Fig. 6). The infection rate also differed among years; the infection rate for the year 2000 (β_reference_) was lower than the other years: 1999 (0.844 ± 0.069), 2001 (0.732 ± 0.089), and 2002 (0.799 ± 0.074; Fig. 6). The infection rate in the control area (β_reference_) was 2.4 to 2.5 times higher than Mallard Road (0.218 ± 0.076) and the New Area (0.221 ± 0.076) and 10.5 times higher than Nauyaug Point (0.051 ± 0.022; Fig. 7). The infection rate was also 13 to 24 times higher for adults than for juveniles depending on the area (Fig. 7).

### Developmental rate

The developmental rate (α) was best modeled as a function of *B*. *burgdorferi* infection status (model A01 in Table 3). The developmental rate was 1.6 times higher for *B*. *burgdorferi*-infected juveniles (1.000 ± 0.001) than for uninfected juveniles (0.643 ± 0.028). There was no support that the developmental rate depended on sex, the annual area-specific burden of infected nymphs (BIN), the annual area-specific tick-induced blood loss in the mouse population (Blood), or the annual area-specific tick burden in the mouse population (Tick.y) (models C01 to C06 have less support than model A01 in Table 3).

### Delta transition

The delta transition (δ) was best modeled as a constant (model A01 in Table 3). There was no support that the delta transition depended on area, month, or sex (models B01 to B04 have less support than model A01 in Table 3).

The results were qualitatively the same for the data set where the adults with the unknown infection state were classified as infected except that there was no support that the infection rate differed among years.

## Discussion

### *B*. *burgdorferi* infection and survival of *P*. *leucopus* mice

Human Lyme disease sufferers are beset with debilitating and often lifelong symptoms including arthritis, joint pain, facial palsy and chronic fatigue [16]. Human longevity would doubtlessly be reduced by *B*. *burgdorferi* infections without the comforts of modern society and western medicine. By contrast, the present study found no evidence that *B*. *burgdorferi* infection reduced the survival of one of its most important reservoir hosts, the white-footed mouse, *P*. *leucopus*. Thus one important, if obvious, conclusion from this study is that our anthropocentric view of Lyme disease pathology is unlikely to be a reliable guide to understanding the population dynamics of this zoonotic disease in nature.

A recent review on the evolutionary ecology of Lyme disease concluded that there is no direct evidence that *Borrelia burgdorferi* s. l. reduces the fitness of its reservoir hosts [16]. Our results are in agreement with this review and previous studies investigating the effect of *B*. *burgdorferi* infection on the fitness of *P*. *leucopus* mice [32, 50]. A two-year CMR study conducted by Hoffmeister et al. [32] in Maryland found no significant effect of *B*. *burgdorferi* infection on the survival of *P*. *leucopus* mice. In another study where *P*. *leucopus* mice were experimentally infected with *B*. *burgdorferi*-infected nymphs, Schwanz et al. [50] found no effect of *B*. *burgdorferi* infection on a variety of rodent running performance measures and blood cell counts over a period of six weeks following the initial infection. Numerous studies have kept experimentally infected rodents in the laboratory over long periods of time (months or years) to quantify mouse-to-tick transmission of *B*. *burgdorferi* [25, 31, 34, 35, 41, 51]. None of these studies have ever reported any acute or chronic infection-related mortality events. Similarly, a recent long-term CMR study on *B*. *garinii* infections in one of its natural sea bird hosts, the black-legged kittiwake, found no effect of this avian *Borrelia* pathogen on host survival [11]. Thus with the exception of one study that found that experimentally infected infant (but not adult) *P*. *leucopus* mice suffered from carditis and arthritis [52], there is no direct evidence that *B*. *burgdorferi* pathogens reduce the survival and/or reproductive success of their natural reservoir hosts. In contrast, the recently discovered association between a genetically polymorphic innate immune receptor in wild bank voles and *B*. *afzelii* infection led Raberg and colleagues to suggest that *Borrelia* pathogens are currently exerting selection on their rodent reservoir hosts [53, 54]. Thus the work by Raberg et al. [53, 54] suggests that *Borrelia* pathogens reduce rodent fitness in the field whereas the study by Hofmeister [32] and the present study have found no evidence that *Borrelia* infection reduces rodent survival under natural conditions. One explanation suggested by Raberg et al. [53] is that *Borrelia* exerts such low levels of selection that they are very difficult to detect in a field study. Thus whether *B*. *burgdorferi* reduces the fitness of wild rodents remains an open question.

The natural history of Lyme disease should select *B*. *burgdorferi* spirochetes to establish an avirulent, chronic infection in the reservoir host. *B*. *burgdorferi* depends on the mobility of the reservoir host to achieve transmission because its tick vector, *I*. *scapularis*, is a sit-and-wait predator that depends on passing hosts to obtain a blood meal [55]. Thus if *B*. *burgdorferi*-induced pathology reduced mobility in important reservoir hosts like *P*. *leucopus*, the pathogen would have fewer opportunities for transmission from infected mice to questing larval ticks. In addition, the strong seasonal asynchrony of nymphal and larval ticks in the Northeastern United States means that *B*. *burgdorferi* has to spend a considerable amount of time in its rodent reservoir host (sometimes several months) before achieving transmission to the next generation of larval ticks [16, 17]. A recent theoretical study investigating the seasonal asynchrony of nymphs and larvae, found that the persistence and prevalence of *B*. *burgdorferi* was highly sensitive to rodent mortality rates [56]. This analysis suggests that tick phenology and seasonal asynchrony of transmission in the Northeastern United States selects for *B*. *burgdorferi* genotypes that establish a non-virulent but chronic infection in its rodent reservoir host [56].

### Limitations of the present study

One of the limitations of the present study was the lack of replication of the control treatment. In addition, there were substantial pre-existing differences in tick density among the four sites before the acaricide treatment (see below). The fact that many of the explanatory variables are correlated (e.g. month, tick burden, and *Borrelia* infection) further complicates the task of separating cause and effect. Most importantly, our conclusions are restricted to the set of candidate models (Table 3). It is possible that an alternative set of candidate models would have led to different conclusions.

### Differences in tick burden and *B*. *burgdorferi* prevalence among areas

Our analyses of the spatial and temporal variation in tick burden and *B*. *burgdorferi* prevalence were consistent with the conclusions of Dolan et al. [38]. The acaracide treatment suppressed the late summer larval peak in 6 of 11 combinations of area and year (Fig. 2). Furthermore, the tick burden and the proportion of *B*. *burgdorferi*-infected mice were substantially higher in the control area than the acaricide-treated areas (Figs. 2 and 3). However, we point out that there were substantial pre-existing differences in tick density among areas. For example, the mean tick burden in Nauyaug Point was almost 15 times lower than the control area (Fig. 2) before the application of the acaricide treatment [38]. Nauyaug Point is a highly exposed, windswept habitat that is unfavourable to ticks whereas the control area was located in the undeveloped centre of the island. Thus differences in tick burden and *B*. *burgdorferi* prevalence among areas were caused by a combination of pre-existing differences in tick density and the acaricide treatment. This naturally occurring spatial variation in tick burden and the force of *B*. *burgdorferi* infection reiterates the importance of using replicate control and treatment plots.

### *Ixodes* tick infestation and host survival

We found no effect of the burden of immature *I*. *scapularis* ticks on the survival or capture rates of *P*. *leucopus* mice. By contrast, a previous 3-year field survey monitoring the density of *P*. *leucopus* and *I*. *scapularis* ticks, found that persistence time of mice on the grid was positively correlated with tick burden [57], suggesting that tick burden enhanced either the survival rate or (more likely) the recapture rate. Studies on the tick *I*. *uriae* have demonstrated a negative effect on the survival and population growth rate of their sea bird hosts [58, 59]. In general there is a great deal of literature on the costs of ectoparasites (fleas, ticks, mites) in birds [60, 61] and rodents [62–64].

Adult mice had a higher tick burden and a higher prevalence of *B*. *burgdorferi* infection than juvenile mice (Table 2) and this observation is consistent with a number of other field studies on wild rodents [32, 65, 66]. Adult male mice had higher tick burdens than adult female mice and this pattern has been found in numerous field studies on *Ixodes* ticks and wild rodents [42, 43, 57, 67–70]. The higher tick burden in males also provides a plausible explanation why the proportion of infected adult mice was higher in males than females. However, our CMR models found no support for a sex-specific difference in the rate of infection (Table 3). One possible explanation for the higher proportion of infected adult mice is that these individuals have the highest recapture rates (see below). Correcting for differences in encounter rates between infected and uninfected individuals is important for obtaining unbiased estimates of disease prevalence [71].

### *B*. *burgdorferi* infection and the recapture rate of *P*. *leucopus* mice

Parasites and pathogens often influence the behavior of their hosts [6], which can have important consequences for the recapture rates. CMR studies of other zoonotic diseases have found that pathogens influence the recapture rates of their reservoir hosts [7–9]. Studies that do not use CMR statistical methods to account for parasite-induced changes in detection probability will come to the wrong conclusions about whether parasites influence host survival. For example, if parasitized individuals are more likely to be encountered than healthy individuals, studies that do not account for this detection bias would wrongly conclude that parasites enhance host survival. Thus to obtain unbiased estimates of whether the pathogen affects host survival, it is critical to consider whether the parasite influences the host recapture rate.

In the present study, we found no effect of *B*. *burgdorferi* infection on recapture rates of adult females but in contrast, *B*. *burgdorferi*-infected adult males were twice as likely to be recaptured than susceptible adult males (Fig. 5). Compared to female recapture rates, the recapture rates of susceptible and *B*. *burgdorferi*-infected males were 35% lower and 25% higher, respectively (Fig. 5). We expected male *P*. *leucopus* mice to have lower recapture rates because they tend to have larger natal dispersal distances and larger adult home range sizes than females [72, 73]. Interestingly, the field study by Hofmeister et al. [32] found that infected *P*. *leucopus* mice had a median lifespan (176 days) that was 12.8% longer than uninfected mice (156 days). However, because the authors did not correct for detection bias, a higher recapture rate of the infected mice may have caused their apparent longer lifespan.

The cause and effect relationship between *B*. *burgdorferi* infection and increased recapture rates in male mice could work in both directions. *B*. *burgdorferi* infection could increase the recapture rate in males by changing their behavior. Alternatively, males with behaviors that result in high recapture rates are more likely to become infected with *B*. *burgdorferi*. Regardless of the chain of causality, the present study illustrates the importance of using appropriate CMR statistical methods to test whether a given pathogen reduces survival in the reservoir host. Statistical approaches that do not correct for pathogen-induced variation in recapture rates will produce biased estimates of pathogen virulence on host survival.

### *B*. *burgdorferi* infection rate in *P*. *leucopus* mice

Our multi-state CMR approach allowed us to test which factors influenced the *B*. *burgdorferi* infection rate of *P*. *leucopus* mice. Our study found that the infection rate of adult mice in the control site was 2.4 to 2.5 times higher than Mallard Road and the New Area and 10.5 times higher than Nauyaug Point (Fig. 7). Thus the acaracide treatments in combination with pre-existing differences in tick density caused the 10-fold difference in the infection rate of adult mice among the four areas. Dolan et al. [38] used the proportion of infections among naive young of the year mice as an index of the infection rate, and the ratios of this index among the different areas were qualitatively very similar to the present study.

As expected, the infection rate of adult mice decreased over the course of the summer (Fig. 5) reflecting the phenology and the peak density of *I*. *scapularis* nymphs, which are more common at the start than the end of the summer (Fig. 2). There was no support that the infection rate depended on the area:month interaction (model D08 in Table 3) suggesting that the acaricide treatment had no effect on the seasonal decline in the infection rate. As expected, the infection rate was an order of magnitude higher for adults than for juveniles (Fig. 7). Juvenile *P*. *leucopus* mice have lower infection rates than adults because they have lower tick burdens (Table 2) and because they develop so fast (3 to 4 weeks; [73]) that they generally reach adulthood before acquiring an infection. The finding that the year 2000 had a lower infection rate resulted from coding the 85 mice with unknown infection status as susceptible because the effect of year disappeared after recoding these mice as infected. The observation that infected juveniles had faster development than susceptible juveniles was caused by grouping juveniles and sub-adults into a single category. Infected juveniles were older sub-adults that inevitably reached adulthood by the next sampling occasion, whereas the susceptible juveniles were true juveniles that first developed into sub-adults before reaching the adult stage.

### The burden of infected nymphs and the *B*. *burgdorferi* infection rate

Characterizing the relationship between vector abundance and the infection rate of reservoir hosts is critical for understanding the epidemiology of vector-borne diseases. Previous field studies have linked the density of questing ticks in the field to measures of tick burden on the reservoir host [57, 74] and the next step is to relate tick burden to the *B*. *burgdorferi* infection rate. Thus one key objective of the present study was to model the infection rate as a quantitative function of the relevant tick burden variables that can be used in future theoretical models. As expected, increasingly relevant tick burden variables received increasingly stronger support as descriptions of the infection process (Table 3): monthly immature tick burden (model D10) < annual immature tick burden (model D07) < nymphal burden (model D04) < burden of infected nymphs (model D03). The burden of infected nymphs (BIN) gave the best quantitative description of the infection process (model D03 in Table 3). This quantitative model received almost as much support as the generic model where infection rate depended on the categorical factors of year, month and area (model D01 in Table 3). The BIN corrected our annual estimates of the nymphal burden by the proportion of larvae that acquired the spirochete from infected *P*. *leucopus* mice the previous year. Thus our modeling exercise picked up the time-lagged contribution of infected *P*. *leucopus* mice in the previous year to the *B*. *burgdorferi* infection rate of the mouse population in the current year. This result is not trivial because larval ticks feed on a community of reservoir hosts [33, 37, 75, 76] that could easily swamp the contribution of *P*. *leucopus*. A previous field study in Connecticut, using a reservoir host vaccination approach, suggested that only 27% of infected nymphs acquired the spirochete from *P*. *leucopus* mice [77]. In the present study, we estimate that ~35% of larval ticks obtained their blood meals from *P*. *leucopus* mice (S2 File), assuming that there are no other competent reservoir hosts on Mason’s Island. Thus the present study confirms that alternative hosts play an important role in the maintenance of Lyme disease [33, 37, 75, 76] on Mason’s Island. Future studies should model the relationship between the density of infected ticks and infection risk as this function is critical to understanding the epidemiology of Lyme disease and to developing prevention strategies [78].

### *B*. *burgdorferi* infection rate estimates in *P*. *leucopus* mice

Our infection rates (defined as the probability that a mouse acquired the infection after surviving for 30 days) ranged from 0.051 to 0.535 (S3 File). Bunnikis et al. [30] used serology to characterize the seasonal infection dynamics of *B*. *burgdorferi* in a population of *P*. *leucopus* in Connecticut and estimated an infection rate of 0.2 cases/mouse/week, which corresponds to 0.590 cases/mouse/month (1 -(1–0.2 cases/mouse/week)^4 weeks/month^). Using a different measure of the infection process (number of new infections/number of mouse-days), Hofmeister et al. [32] estimated an incidence rate of 5.9 infections per 1000 mouse-days in a population of *P*. *leucopus* in Maryland. By contrast, the study by Bunnikis et al. [30] observed an incidence rate of 29 infections per 1000 mouse-days, and our study estimated an incidence of 97 infections/1988 captures = 48.8 infections per 1000 mouse-days. The eight-fold variation in the incidence rate among studies may be caused by different detection methods for *B*. *burgdorferi* infection (culture of tissue samples in BSK media versus serology and antibody-based methods). Alternatively, the variation in the incidence rate may represent true differences in the force of infection among populations of *P*. *leucopus*. In any event, our estimate of the incidence rate is considerably higher than what has been reported in the literature so far.

### Survival rate estimates for *P*. *leucopus* mice

Our monthly survival estimates of adult *P*. *leucopus* mice in the control area ranged from 0.552 to 0.875. These estimates were lower than a recently published study on a *P*. *leucopus* population in northeastern Oklahoma (monthly survival rate ranged from 0.760 to 0.937 and was calculated from the quarterly survival rate, which ranged from 0.440 to 0.822) [79]. There was considerable tag loss in the current study (13.4%), which would result in underestimates of mouse survival. Workers should consider using double tagging methods or transponders to avoid the problem of tag loss in future studies [80, 81].

Survival was higher in Nauyaug Point than the other three areas (Fig. 4). We point out that our survival estimates represent ‘apparent survival’ estimates, which are biased low by permanent migration from the study area. One explanation for the higher ‘apparent survival’ rates on Nauyaug Point is that opportunities for permanent migration from an island isthmus are more limited compared to the other three areas, which are in the centre of the island. The observation that the survival rates of the control area were similar to Mallard Road and the New Area suggests that the acaricide treatment did not affect rodent survival. This confirms the observation by Dolan et al. [38] that neither the food in the acaricide-treated bait boxes nor the food in the Sherman traps affected the mouse population size.

We had expected survival to be lower during the winter due to inclement weather and/or limited food resources. However, we found that survival over the 8-month sampling hiatus (October to April) was similar to the August survival rate and higher than the survival rates at other times in the summer (Fig. 4). Schug et al. [82] proposed that adult mortality increased in spring and summer due to breeding-associated intraspecific competition. However, other studies have found that *P*. *leucopus* mice actually stop breeding in the summer in response to infection with helminth parasites [83]. Long-term field studies on botfly macroparasites in *P*. *leucopus* populations have shown that infested mice live longer than uninfested mice [84, 85]. The hypothesized mechanism is that the botfly parasite shifts resources from mouse reproduction to mouse survival. Thus host-parasite interactions can lead to complex trade-offs between life history traits [86]. The present study did not test whether *B*. *burgdorferi* influenced mouse reproduction. For the sake of simplicity, we also remained within the one parasite—one host framework. Future studies should investigate how *B*. *burgdorferi* infection interacts with other parasites to influence the survival and reproduction of their rodent reservoir hosts.

## Conclusions

We found no evidence that *B*. *burgdorferi* s. s., the causative agent of Lyme disease in North America, reduced the survival of one of its most important reservoir hosts, the white-footed mouse, *P*. *leucopus*. Consideration of the epidemiology of Lyme disease suggests that *B*. *burgdorferi* should be under strong selection to establish chronic but non-virulent infections in its natural reservoir hosts. *B*. *burgdorferi* infection was associated with higher recapture rates in male mice but not in female mice. The infection rates decreased over the course of the transmission season and were an order of magnitude higher for adult mice compared to juvenile mice. The estimates of *P*. *leucopus* survival and *B*. *burgdorferi* infection risk will be useful for parameterizing future theoretical studies examining the epidemiology of Lyme disease and strategies of disease control in this important reservoir host.

## Supporting Information

S1 File Generalized linear model analysis of the effect of the acaricide treatment on tick burden and *Borrelia burgdorferi* prevalence in *Peromyscus leucopus* mice. (DOCX)

S2 File Calculation of the burden of infected nymphs and the proportion of incompetent hosts that feed larval ticks. (DOCX)

S3 File Model-averaged parameter estimates for the five parameters of the multi-state capture-mark-recapture models: (1) survival rate, (2) recapture rate, (3) infection rate, (4) development rate, and the (5) delta transition rate. (DOCX)
