# Supplementary material for: The Lyme Disease Pathogen Has No Effect on the Survival of Its Rodent Reservoir Host
Source: PLoS One. 2015 Feb 17;10(2):e0118265. doi: 10.1371/journal.pone.0118265 (PMC4331372; doi:10.1371/journal.pone.0118265)
Supplement: S1 File — (DOCX) [file pone.0118265.s001.docx]

Supporting Information file S1

**BACKGROUND:**

Dolan et al. [[38](#_ENREF_38)] presented the data for the first three years (1999 to 2001) and for three of the four areas (Control Area, Nauyaug Point, New Area but not Mallard Road). We re-analysed the data using generalized linear models to confirm that there were differences in *Ixodes scapularis* tick burden and the proportion of *Borrelia burgdorferi*-infected *Peromyscus leucopus* mice among areas, months, and years.

**STATISTICAL METHODS:**

**Effect of acaricide treatment on tick burden and *B. burgdorferi* prevalence in *P. leucopus* mice:** We used generalized linear models (GLMs) with a negative binomial error function to analyse the tick burden data [[41](#_ENREF_41), [42](#_ENREF_42)]. We used GLMs with a binomial error function to analyse the proportion of *B. burgdorferi*-infected *P. leucopus* mice data. Area was treated as a fixed factor with four levels: Control Area, Mallard Road, Nauyaug Point, and New Area. Month was treated as a fixed factor with five levels: May, June, July, August, and September. Year was treated as a linear covariate and the years 1999, 2000, 2001, 2002 were converted to the integers 1, 2, 3, 4, respectively. We ranked models according to their AIC score and examined the parameter estimates to make inferences about the explanatory factors of interest.

**RESULTS:**

**Effect of acaricide treatment on tick burden in *P. leucopus* mice:** The negative binomial error function provided an excellent fit to the tick burden data as indicated by the fact that the ratio of the residual deviance and the residual degrees of freedom was close to 1.00 (ratio = 1627.1/1821 = 0.8935 for model 1 in Table S1.1). The tick burden in the control area showed the typical phenology of immature *Ixodes scapularis* ticks in the Northeastern United States with the nymphal peak occurring in the months of May, June, and July and the larval peak occurring in August and September (Figure S1.1). For tick burden, the most parsimonious model included the main effects of area, year, and month as well as the area:month and the year:month interactions (model 1 in Table S1.1). The three top models had 100% of the support and all three models included the area:month and the year:month interactions (models 1, 2, and 3 in Table S1.1). Support for the area:month interaction confirmed our visual observation that the larval peak was often suppressed in the acaricide-treated areas but not in the control area (Figure S1.1). Similarly, support for the year:month interaction confirmed our visual observation that the acaricide-treatment suppressed the larval peak in some but not all years (Figure S1.1). The tick burden was lower in the acaricide-treated areas than the control area and this difference was particularly pronounced during the months of the larval peak (August and September) (Figure S1.1).

**Effect of acaricide treatment on *B. burgdorferi* prevalence in *P. leucopus* mice:** The binomial error function provided a good fit to the proportion of *B. burgdorferi*-infected mice data as indicated by the fact that the ratio of the residual deviance and the residual degrees of freedom was close to 1.00 (ratio = 2173.793/1706 = 1.274205 for model 1 in Table S1.2). For the proportion of *B. burgdorferi*-infected mice, the most parsimonious model included the main effects of area, year, month, and the area:year interaction (model 1 in Table S1.2). The two top models had 99.91% of the support and both models included the area:year interaction (models 1 and 2 in Table S1.2). Support for the area:year interaction confirmed our visual observation that the proportion of *B. burgdorferi*-infected mice declined in Nauyaug Point over the course of the study (Figure S1.2). The proportion of *B. burgdorferi*-infected mice was lower in the acaricide-treated areas, and higher in the months of June, July, and August than in the months of May and September (Figure S1.2).

Table S1.1. The burden of immature *Ixodes scapularis* ticks (larvae and nymphs) on *Peromyscus leucopus* mice was modelled as a function of three explanatory variables: area (A), year (Y), and month (M) using a generalized linear model with a negative binomial error function. Area is a fixed factor with four levels: Control Area, Mallard Road, Nauyaug Point, and New Area. Year is a covariate where the years 1999, 2000, 2001, and 2002 were converted to the integers 1, 2, 3, and 4, respectively. Month is a fixed factor with five levels: May, June, July, August, and September. The 16 models were ranked according to their AIC score. Each model was assigned a unique model identification number (Model ID) and the model structure shows the factors and interaction terms included in that particular model. Also shown for each model are the numbers of parameters (# Param), the residual degrees of freedom (DF_resid_), the residual deviance (Dev_resid_), the AIC score (AIC), the difference in AIC between each model and the best model (ΔAIC), and the weight of each model (Weight).

| Model ID | Model Structure | # Param | DF_resid_ | Dev_resid_ | AIC | ΔAIC | Weight |
| --- | --- | --- | --- | --- | --- | --- | --- |
| 1 | A + Y + M + A:M + Y:M | 25 | 1836 | 1640.3 | 6932.3 | 0.0 | 0.568 |
| 2 | A + Y + M + A:Y + A:M + Y:M + A:Y:M | 40 | 1821 | 1627.1 | 6934.0 | 1.7 | 0.243 |
| 3 | A + Y + M + A:Y + A:M + Y:M | 28 | 1833 | 1639.1 | 6934.5 | 2.2 | 0.189 |
| 4 | A + Y + M + A:Y + A:M | 24 | 1837 | 1640.1 | 6954.5 | 22.2 | 0.000 |
| 5 | A + Y + M + Y:M | 13 | 1848 | 1642.5 | 6954.9 | 22.6 | 0.000 |
| 6 | A + Y + M + A:Y + Y:M | 16 | 1845 | 1641.6 | 6955.4 | 23.1 | 0.000 |
| 7 | A + Y + M + A:M | 21 | 1840 | 1641.8 | 6956.1 | 23.8 | 0.000 |
| 8 | A + Y + M + A:Y | 12 | 1849 | 1645.2 | 6981.3 | 49.0 | 0.000 |
| 9 | A + M | 8 | 1853 | 1648.1 | 6983.9 | 51.6 | 0.000 |
| 10 | A + Y + M | 9 | 1852 | 1648.0 | 6985.8 | 53.5 | 0.000 |
| 11 | A | 4 | 1857 | 1664.5 | 7224.4 | 292.1 | 0.000 |
| 12 | A + Y | 5 | 1856 | 1663.2 | 7224.8 | 292.5 | 0.000 |
| 13 | Y + M | 6 | 1855 | 1683.1 | 7622.1 | 689.8 | 0.000 |
| 14 | M | 5 | 1856 | 1683.5 | 7622.1 | 689.8 | 0.000 |
| 15 | 1 | 1 | 1860 | 1669.8 | 7769.8 | 837.5 | 0.000 |
| 16 | Y | 2 | 1859 | 1669.9 | 7771.6 | 839.3 | 0.000 |

Table S1.2. The proportion of *Borrelia burgdorferi*-infected *Peromyscus leucopus* mice was modelled as a function of three explanatory variables: area (A), year (Y), and month (M) using a generalized linear model with a binomial error function. Area is a fixed factor with four levels: Control Area, Mallard Road, Nauyaug Point, and New Area. Year is a covariate where the years 1999, 2000, 2001, and 2002 were converted to the integers 1, 2, 3, and 4, respectively. Month is a fixed factor with five levels: May, June, July, August, and September. The 16 models were ranked according to their AIC score. Each model was assigned a unique model identification number (Model ID) and the model structure shows the factors and interaction terms included in that particular model. Also shown for each model are the numbers of parameters (# Param), the residual degrees of freedom (DF_resid_), the residual deviance (Dev_resid_), the AIC score (AIC), the difference in AIC between each model and the best model (ΔAIC), and the weight of each model (Weight).

| Model ID | Model Structure | # Param | DF_resid_ | Dev_resid_ | AIC | ΔAIC | Weight |
| --- | --- | --- | --- | --- | --- | --- | --- |
| 1 | A + Y + M + A:Y | 12 | 1734 | 2207.9 | 2231.9 | 0.0 | 0.5976 |
| 2 | A + Y + M + A:Y + Y:M | 16 | 1730 | 2200.7 | 2232.7 | 0.8 | 0.4014 |
| 3 | A + Y + M + A:Y + A:M | 24 | 1722 | 2197.9 | 2245.9 | 14.0 | 0.0005 |
| 4 | A + Y + M + A:Y + A:M + Y:M | 28 | 1718 | 2192.1 | 2248.1 | 16.2 | 0.0002 |
| 5 | A + Y + M | 9 | 1737 | 2231.6 | 2249.6 | 17.7 | 0.0001 |
| 6 | A + M | 8 | 1738 | 2234.0 | 2250.0 | 18.1 | 0.0001 |
| 7 | A + Y + M + Y:M | 13 | 1733 | 2224.6 | 2250.6 | 18.7 | 0.0001 |
| 8 | A + Y + M + A:Y + A:M + Y:M + A:Y:M | 40 | 1706 | 2173.8 | 2253.8 | 21.9 | 0.0000 |
| 9 | A + Y + M + A:M | 21 | 1725 | 2220.6 | 2262.6 | 30.7 | 0.0000 |
| 10 | A + Y + M + A:M + Y:M | 25 | 1721 | 2214.2 | 2264.2 | 32.3 | 0.0000 |
| 11 | A + Y | 5 | 1741 | 2258.0 | 2268.0 | 36.1 | 0.0000 |
| 12 | A | 4 | 1742 | 2260.7 | 2268.7 | 36.8 | 0.0000 |
| 13 | Y + M | 6 | 1740 | 2348.6 | 2360.6 | 128.6 | 0.0000 |
| 14 | M | 5 | 1741 | 2352.6 | 2362.6 | 130.7 | 0.0000 |
| 15 | Y | 2 | 1744 | 2380.4 | 2384.4 | 152.5 | 0.0000 |
| 16 | 1 | 1 | 1745 | 2385.1 | 2387.1 | 155.2 | 0.0000 |

Figure S1.1. Average monthly burden of immature *Ixodes scapularis* ticks (larvae and nymphs) on *Peromyscus leucopus* mice showed a seasonal pattern over the four years of the study (1999, 2000, 2001, and 2002) for each of the four areas (Control Area, Mallard Road, Nauyaug Point, New Area). Tick burden has units of number of ticks per mouse. There are five sampling months within each year (May, June, July, August, September). The size of the circle is proportional to the number of mice on which the average is based (range: 1 to 64). The properties in the New Area were not sampled in 1999.

Figure S1.2. Monthly prevalence of *Borrelia burgdorferi*-infected *Peromyscus leucopus* mice varied over the four years of the study (1999, 2000, 2001, and 2002) for each of the four areas (Control Area, Mallard Road, Nauyaug Point, New Area). The prevalence is a proportion and therefore does not have units. There are five sampling months within each year (May, June, July, August, September). The size of the circle is proportional to the number of mice on which the prevalence is based (range: 1 to 64). The properties in the New Area were not sampled in 1999. There is no estimate for September 2000 because the mice were not tested for *B. burgdorferi* infection.
