# Supplementary material for: The Lyme Disease Pathogen Has No Effect on the Survival of Its Rodent Reservoir Host
Source: PLoS One. 2015 Feb 17;10(2):e0118265. doi: 10.1371/journal.pone.0118265 (PMC4331372; doi:10.1371/journal.pone.0118265)
Supplement: S2 File — (DOCX) [file pone.0118265.s002.docx]

Supporting Information file S2

**Burden of infected nymphs index 1 (BIN_1_):** We need to correct the annual *Ixodes scapularis* nymphal tick burden for the proportion of nymphs infected with *Borrelia burgdorferi* (PIN = proportion of infected nymphs). Dolan et al. [[1](#_ENREF_1)] used nested PCR to estimate the proportion of *B. burgdorferi*-infected questing nymphs in two areas (Control Area and New Area) for two years (2000 and 2001), which is not sufficient data for our purposes (see Table 4 in Dolan et al. [[1](#_ENREF_1)] and Table S2.1). We can approximate the proportion of infected nymphs in year t (PIN_(t)_) with the proportion of infected larvae in the previous year (PIL_(t-1)_). We calculated PIL_(t-1)_ as the proportion of larvae that acquired a *B. burgdorferi* infection after feeding on *Peromyscus leucopus* mice in year t-1 (PIL_PL(t-1)_). For each of the 16 combinations of area and year, we calculated the PIL_PL(t-1)_ as follows:

$${PIL}_{PL\left( t-1 \right)}=\frac{{NIL}_{PL\left( t-1 \right)}}{{TNL}_{PL\left( t-1 \right)}}$$

where NIL_PL(t-1)_ = the number of larvae that acquired a *B. burgdorferi* infection after feeding on *P. leucopus* mice in year t-1 and TNL_PL(t-1)_ = the total number of larvae that fed on *P. leucopus* mice in year t-1. NIL_PL(t-1)_ was calculated by multiplying the larval tick burden (i.e. the tick burdens in the months of August and September) by the infection status of the mouse (0 = uninfected, 1 = infected) and the mouse-to-tick transmission coefficient of *B. burgdorferi*, which is generally assumed to be 0.90 for *P. leucopus* [[2](#_ENREF_2),[3](#_ENREF_3)] and then summing over all *P. leucopus* mice. TNL_PL(t-1)_ was calculated by summing the larval tick burdens (i.e. the tick burdens in the months of August and September) over all *P. leucopus* mice. One advantage of this estimate of the PIL_PL_ is that it accounts for the aggregated distribution of ticks on mice [[4](#_ENREF_4),[5](#_ENREF_5),[6](#_ENREF_6)]. For the first year of the study (1999), we could not calculate the PIL_PL_ value for the previous year (1998) and therefore assumed that PIL_PL(1998)_ = PIL_PL(1999)_. We thus obtained our first estimate of the burden of infected nymphs (BIN_1_) by multiplying the mean annual nymph burden in year ‘t’ by the PIL_PL_ variable in year ‘t-1’. This first estimate of the burden of infected nymphs (BIN_1_) is biased high because it assumes that larvae only feed on *P. leucopus*, which is a highly competent host for *B. burgdorferi*. In reality, many larvae feed on other reservoir hosts that are less competent at becoming infected with *B. burgdorferi* and/or at transmitting the spirochete to feeding larvae.

**Burden of infected nymphs index 2 (BIN_2_):** We need to correct for the fact that not all larvae take their meals from *P. leucopus* hosts. We can correct the PIL_PL_ values by comparing them to the proportion of infected questing nymphs (PIN) the following year as estimated by Dolan et al. [[1](#_ENREF_1)]. Dolan et al. [[1](#_ENREF_1)] estimated the PIN for four combinations of area (Control Area and New Area) and year (2000, 2001) to be 26%, 20%, 24% and 8% (Table S2.1). By contrast, we calculated the PIL_PL_ for the 1999 Control Area, the 2000 Control Area, and the 2000 New Area to be 69.1%, 60.9% and 50.9% (no data for the 1999 New Area; Table S2.1). This discrepancy between the two sets of estimates suggests that the tick larvae are feeding on other hosts that are less competent at transmitting *B. burgdorferi*. The PIN and the PIL_PL_ are related to each other as follows: PIN_(t+1)_ = ƒ_(t)_*PIL_PL(t)_ + (1 – ƒ)_(t)_* PIL_OH(t)_, where ƒ is the fraction of larvae that fed on *P. leucopus* mice, 1 – ƒ is the fraction of larvae that fed on other hosts, PIL_PL_ is the proportion of larvae that acquired the spirochete from *P. leucopus* mice, and PIL_OH_ is the proportion of larvae that acquired the spirochete from other hosts. If we assume that PIL_OH_ = 0, then ƒ_(t)_ = PIN_(t+1)_/ PIL_PL(t)_. By assuming that PIL_PL(1999)_ = PIL_PL(2000)_ = 0.509, we were able to calculate ƒ_(t)_ for each of the four combinations of area and year (Table S2.1). Our estimates of ƒ_(t)_ suggested that ~35.2% of all larvae obtained their blood meal from *P. leucopus* mice in the Control Area. By contrast, in the New Area, 47.2% of all larvae obtained their blood meal from *P. leucopus* mice in 1999 (before the use of acaricides) whereas only 15.7% of all larvae obtained their blood meal from *P. leucopus* mice in 2000 (after these mice were treated with acaricides). As we had limited data (only 3 combinations of area and year in Table S2.1), we calculated our second estimate of the burden of infected nymphs (BIN_2_) by multiplying the BIN_1_ values by 33.3% (the average of the four estimates of ƒ in Table S2.1).

Table S2.1. Proportion of larvae that acquired *Borrelia burgdorferi* after feeding on infected *Peromyscus leucopus* mice (PIL_PL_) and the proportion of infected questing nymphs (PIN) the following year estimated for two sites and two years. The PIL_PL_ was calculated from the larval tick burden on *P. leucopus* mice, the *B. burgdorferi*-infection status of these mice, and assumed a mouse-to-tick transmission coefficient of *B. burgdorferi* of 0.90 [[2](#_ENREF_2),[3](#_ENREF_3)]. Dolan et al. [[1](#_ENREF_1)] estimated the PIN on questing nymphs that were collected by flagging and tested for *B. burgdorferi* infection using nested PCR. The fraction of larvae that had obtained their blood meal from *P. leucopus* mice (ƒ), was calculated by assuming that there was no transmission of *B. burgdorferi* from other hosts to feeding larvae. To calculate ƒ_1999_, we assumed that PIL_1999_ = PIL_2000_ = 0.509.

| Site | Year | PIL_PL_ | Year | PIN | ƒ |
| --- | --- | --- | --- | --- | --- |
| Control | 1999 | 0.691 | 2000 | 0.26 | 0.376 |
| Control | 2000 | 0.609 | 2001 | 0.20 | 0.328 |
| New | 1999 | ND | 2000 | 0.24 | 0.472 |
| New | 2000 | 0.509 | 2001 | 0.08 | 0.157 |

**References**

1. Dolan MC, Maupin GO, Schneider BS, Denatale C, Hamon N, et al. (2004) Control of immature *Ixodes scapularis* (Acari: Ixodidae) on rodent reservoirds of *Borrelia burgdorferi* in a residential community of southeastern Connecticut. Journal of Medical Entomology 41: 1043-1054.

2. Brisson D, Dykhuizen DE (2004) *ospC* diversity in *Borrelia burgdorferi*: different hosts are different niches. Genetics 168: 713-722.

3. LoGiudice K, Ostfeld RS, Schmidt KA, Keesing F (2003) The ecology of host diversity and community composition on Lyme disease risk. Proceedings of the National Academy of Sciences of the United States of America 100: 567-571.

4. Brunner JL, Ostfeld RS (2008) Multiple causes of variable tick burdens on small-mammal hosts. Ecology 89: 2259-2272.

5. Devevey G, Brisson D (2012) The effect of spatial heterogeneity on the aggregation of ticks on white-footed mice. Parasitology 139: 915-925.

6. Randolph SE, Miklisova D, Lysy J, Rogers DJ, Labuda M (1999) Incidence from coincidence: patterns of tick infestations on rodents facilitate transmission of tick-borne encephalitis virus. Parasitology 118: 177-186.
