# Supplementary material for: The Lyme Disease Pathogen Has No Effect on the Survival of Its Rodent Reservoir Host
Source: PLoS One. 2015 Feb 17;10(2):e0118265. doi: 10.1371/journal.pone.0118265 (PMC4331372; doi:10.1371/journal.pone.0118265)
Supplement: S3 File — (DOCX) [file pone.0118265.s003.docx]

Supporting Information file S3

**BACKGROUND:**

We calculated model-averaged parameter estimates for the 42 models in the model selection table (Table 3 in the main manuscript). There are five parameters for the multi-state capture-mark-recapture (CMR) models: (1) survival rate, (2) recapture rate, (3) infection rate, (4) developmental rate, and the (5) delta transition rate. The survival rate (φ) is the probability that a mouse will survive a time interval of 30 days. The recapture rate (p) is the probability that the mouse will be recaptured during a sampling session. The infection rate (β) is the probability that an uninfected mouse will become infected conditional on having survived the previous time interval of 30 days. The developmental rate (α) is the probability that a juvenile mouse will develop into an adult mouse conditional on having survived the previous time interval of 30 days. The delta transition (δ) is the probability that an uninfected juvenile will develop into an infected adult mouse conditional on having survived the previous time interval of 30 days. The delta transition contains two biological transitions: uninfected to infected and juvenile to adult.

Table S3.1 shows the parameter estimates for a given set of explanatory factors. For example, the first row shows the survival rate (φ) of adult, susceptible female mice (stage = A for adult, *Borrelia burgdorferi* (Bb) infection status = S for susceptible, sex = F for female) in the Control area for the year 2000 and the month of May. The survival estimate for this group of mice is 0.552. Also shown are the standard error and the 95% lower and upper confidence limits.

Table S3.1. Parameter estimates for survival rate (φ), recapture rate (p), infection rate (β), developmental rate (α), and the delta transition rate (δ). Parameters are classified according to stage (A = adult, J = juvenile), *Borrelia burgdorferi* (Bb) infection (I = infected, S = susceptible), sex (F = female, M = male), year (1999, 2000, 2001, and 2002), month (May, June, July, August, and September). Shown are the parameter estimates (Est), standard errors (SE), lower (LCL) and upper confidence limits (UCL).

| par | stage | Bb | sex | Area | year | mnth | Est | SE | LCL | UCL |
| --- | --- | --- | --- | --- | --- | --- | --- | --- | --- | --- |
| φ | A | S | F | Cont | 2000 | **May** | 0.552 | 0.050 | 0.454 | 0.646 |
| φ | A | S | F | Cont | 2000 | **June** | 0.593 | 0.056 | 0.480 | 0.697 |
| φ | A | S | F | Cont | 2000 | **July** | 0.608 | 0.056 | 0.495 | 0.710 |
| φ | A | S | F | Cont | 2000 | **Aug** | 0.875 | 0.127 | 0.419 | 0.986 |
| φ | A | S | F | Cont | 2000 | **Sept** | 0.787 | 0.028 | 0.728 | 0.837 |
| φ | A | S | F | **Cont** | 2000 | May | 0.552 | 0.050 | 0.454 | 0.646 |
| φ | A | S | F | **Mal** | 2000 | May | 0.559 | 0.053 | 0.454 | 0.658 |
| φ | A | S | F | **Nau** | 2000 | May | 0.664 | 0.059 | 0.542 | 0.768 |
| φ | A | S | F | **New** | 2000 | May | 0.532 | 0.053 | 0.429 | 0.632 |
| p | A | S | F | Cont | 2000 | **May** | 0.324 | 0.049 | 0.236 | 0.427 |
| p | A | S | F | Cont | 2000 | **June** | 0.418 | 0.052 | 0.320 | 0.522 |
| p | A | S | F | Cont | 2000 | **July** | 0.438 | 0.054 | 0.336 | 0.546 |
| p | A | S | F | Cont | 2000 | **Aug** | 0.192 | 0.040 | 0.125 | 0.283 |
| p | A | S | F | Cont | 2000 | **Sept** | 0.249 | 0.055 | 0.157 | 0.372 |
| p | A | S | F | cont | 2000 | May | 0.324 | 0.049 | 0.236 | 0.427 |
| p | A | S | **M** | cont | 2000 | May | 0.214 | 0.042 | 0.143 | 0.309 |
| p | A | **I** | F | cont | 2000 | May | 0.333 | 0.044 | 0.253 | 0.423 |
| p | A | **I** | **M** | cont | 2000 | May | 0.411 | 0.048 | 0.321 | 0.508 |
| β | J | S | F | **cont** | 2000 | May | 0.042 | 0.021 | 0.016 | 0.110 |
| β | J | S | F | **mal** | 2000 | May | 0.011 | 0.008 | 0.003 | 0.042 |
| β | J | S | F | **nau** | 2000 | May | 0.002 | 0.003 | -0.003 | 0.008 |
| β | J | S | F | **new** | 2000 | May | 0.011 | 0.007 | 0.003 | 0.041 |
| β | A | S | F | **cont** | 2000 | May | 0.535 | 0.095 | 0.353 | 0.708 |
| β | A | S | F | **mal** | 2000 | May | 0.218 | 0.076 | 0.104 | 0.400 |
| β | A | S | F | **nau** | 2000 | May | 0.051 | 0.022 | 0.021 | 0.117 |
| β | A | S | F | **new** | 2000 | May | 0.221 | 0.076 | 0.107 | 0.402 |
| β | A | S | F | cont | 2000 | **May** | 0.535 | 0.095 | 0.353 | 0.708 |
| β | A | S | F | cont | 2000 | **June** | 0.584 | 0.092 | 0.401 | 0.746 |
| β | A | S | F | cont | 2000 | **July** | 0.404 | 0.095 | 0.239 | 0.595 |
| β | A | S | F | cont | 2000 | **Aug** | 0.180 | 0.080 | 0.070 | 0.389 |
| β | A | S | F | cont | 2000 | **Sept** | 0.123 | 0.110 | 0.019 | 0.507 |
| β | A | S | F | cont | **1999** | May | 0.844 | 0.069 | 0.660 | 0.938 |
| β | A | S | F | cont | **2000** | May | 0.535 | 0.095 | 0.353 | 0.708 |
| β | A | S | F | cont | **2001** | May | 0.732 | 0.089 | 0.528 | 0.869 |
| β | A | S | F | cont | **2002** | May | 0.799 | 0.074 | 0.617 | 0.908 |
| α | J | S | F | cont | 2000 | May | 0.643 | 0.028 | 0.587 | 0.696 |
| α | J | **I** | F | cont | 2000 | May | 1.000 | 0.001 | 0.999 | 1.001 |
| δ | J | S | F | cont | 2000 | May | 0.152 | 0.011 | 0.131 | 0.174 |
